# Understanding H-aggregates crystallization induced emissive behavior: insights from theory

**DOI:** 10.1038/s41598-023-39605-5

**Published:** 2023-07-31

**Authors:** Huixue Li, Lingling Lv, Kun Yuan, Sujuan Pan, Zhifeng Li

**Affiliations:** School of Chemical Engineering and Technology, Tianshui Normal University, Tianshui, 741001 Gansu China

**Keywords:** Fluorescence spectroscopy, Computational chemistry

## Abstract

We conducted a theoretical investigation into how the molecular stacking effect impacts the photophysical properties in solid phases. Our findings indicated that in the aggregated state, the out-of-plane distorted vibration and imidazole ring stretching vibration of triimidazo-[1,3,5] triazinethe are significantly suppressed, which decreased the Huang-Rhys factor and the corresponding reorganization energy of the photophysical process, as a result, this restricted intramolecular motions and dissipation pathways of excess energy in the excited state, therefore, aggregation induced enhancement emission (AIEE) was found for the title compound from dichloromethane solution to solid state. Analysis of the emission spectrum through discrete spectral lines revealed that the main peak was affected by the vibrational modes with lower frequencies, while the middle-frequency modes influenced the shoulder peak. Furthermore, the predicted intersystem crossing rate (*k*_iosk_) and reverse intersystem crossing rate (*k*_risc_) using Marcus theory confirmed that an electron can successfully shift from its S_1_ state to the T_1_ state, however, the reverse T_1_ → S_1_ process can not come into being due to very small *k*_risc_ (10^–6^–10^–9^ s^–1^), therefore the phosphorescence can be observed. At last, we explored the influence of charge transfer process of the title compound, our theoretical data declared this process can be ignored due to its low transfer rate.

## Introduction

Due to their exceptional luminosity efficiency and vibrant illumination colors, organic light-emitting materials have been organic light-emitting materials have been found extensive applications in various fields, including chemical detection, biological sensing and imaging, and photovoltaic devices^1–7^. However, the currently available fluorescent materials, which possess a macrocyclic aromatic conjugation system, face a significant challenge in research and practical applications. These organic light–emitting materials tend to pack tightly in aggregates due to their planar polycyclic aromatic structure, the π–π stacking interaction between molecules in condensed state leads to the formation of detrimental excimers at the excited state, resulting in well–known the aggregation–caused quenching (ACQ) effect^5,8,9^. This ACQ effect theoretically and practically reduces the fluorescence quantum yield of the material. Fortunately, not all organic fluorescent materials exhibit the limitations of ACQ. In fact, there is an intriguing phenomenon known as aggregation-induced emission (AIE), and they have been discovered in numerous molecular systems^4,8,10–14^. AIE allows these molecules to exhibit more efficient light emission when they aggregate, in the case of these materials, their fluorescence is either absent or extremely weak when in dilute solution, nevertheless, their luminescent intensity significantly enhances when they are in a gathered or solid state. Actually, their fluorescence quantum yield increases by several orders of magnitude when in the solid state as compared to that in a solution^7^. Up to now, it has been proved that numerous planar ACQ chromophores can be transformed into AIE materials by combination of other AIE-active unit (for instance tetraphenylethene)^15,16^.

The emergence of the AIE effect has successfully overcome the limitations of the traditional fluorescent molecules utilized in photoelectric devices. This effect offers a remarkable improvement in the luminescent performance of materials and enhances the detection sensitivity. Consequently, it has garnered significant attention from both the scientific community and industry, becoming a prominent area of research in chemistry and materials science. As a result, an increasing number of studies in various fields including chemistry, biology, and materials have been conducted, leveraging the advantages provided by the AIE effect^17–22^. Besides the experimental observation, a variety of theoretical investigations have also been done to elucidate the AIE mechanisms and predict new AIE materials^8,9,23–25^. The mechanism of restricting intramolecular motion (RIM)—which involves limiting both intramolecular rotation and vibration—has been widely accepted due to its strong support from experimental evidence^9,26,27^.

J-aggregation is another mechanism proposed for AIE luminescent materials^5,28^, it involves the head–tail overlap of fluorophores when both the molecules aggregate with the assistance of solvents or supermolecular self-organization. During this aggregation process, the spatial arrangement of molecules can effectively diminish the strong π–π interaction between aromatic compounds and hinder the formation of excimers, resulting in enhanced fluorescence. However, it is worth noting that not every J-aggregation possesses AIE activity^9^.

While it is generally accepted that H-aggregates are non-emissive materials, there are exceptions to the rule. Some H-aggregates have been found to exhibit strong fluorescence and high efficiency^29,30^. In a recent study, Cariati and colleagues^31^ reported on a pure organic molecule called cyclic triimidazole, which exhibits H-aggregation. Interestingly, this molecule exhibited weak luminescence in solution but displayed robust luminescence when in a crystalline form. To investigate its emissive behavior, the research team employed time-resolved emission spectroscopy and observed both fluorescence and phosphorescence phenomena experimentally. Notably, the compound exhibited an ultralong phosphorescence lifetime of up to one second, even at room temperature in air. While the researchers attributed the luminescent phenomenon to crystallization-induced emission based on a combination of experimental and theoretical calculations, but the actions of excited processes involved all sorts of radiative and nonradiative decay, intersystem crossing (ISC) and reverse intersystem crossing (RISC) rates between ground state (S_0_), first excited state (S_1_) and first triplet state (T_1_), still remained undiscovered, the lack of quantitative discussion was flaw in their paper.

Currently, numerous researchers have made significant contributions to theoretical modeling and approaches related to AIE. Shuai et al. developed an algorithm to be called time-dependent density matrix renormalization group at zero and finite temperature to compute the linear absorption and fluorescence spectra of molecular gathered state, this method can provide an accurate and efficient means to calculate the spectrum of molecular aggregation^32^. Using the QM/MM model, they found the concomitant relationship between the descriptors (γ, β) and phosphorescence efficiency and lifetime, it was also revealed that the organic compounds containing n/π-groups were favorable for the room–temperature phosphorescence in organic molecules with high efficiency and long–lived afterglow synchronously^33^. Presti et al. used a recently developed excited–state electrostatic embedding model to elucidate the enhanced emission in fluorenone compound, they focused on a single-molecule process only, and found it played an important role to enhance fluorescence that the electrostatic field was induced by the crystalline environment at the excited state, Moreover, they observed a substantial bathochromic shift compared with emission in dilute solution^34^. Tang et al. introduced benzoyl or benzyl to a planar chromophore to create AIE luminogens, they analyzed and depicted the operating mechanism of these new AIE luminogens, in which the structural rigidification of these fused-ring aromatic compounds were the major factor to be responsible for their AIE effect^22^. Liang and Shuai et al. proposed that resonance Raman spectroscopy (RRS) was employed to research the microscopic mechanism of AIE, owing to the RRS amplitude is proportional to frequency times the vibrational relaxation energy of mode, therefore RRS was a direct way to confirm the AIE mechanism^35^. By the computational study, Basak et al. explained the unique fluorescence property of the H-aggregated naphthalene diimide (NDI) derivative, it was found that the S_2_ → S_0_ transition was responsible for the fluorescence when the S_1_ was a dark state, obviously it violated Kasha's rule and was accountable for the unique fluorescence properties of this type of NDI molecule^29^.

In this study, we aimed to deepen AIE understanding of the luminescent mechanism about cyclic triimidazole by a comprehensive theoretical investigation. Firstly, we optimized the geometries of the compound in gas phase, solution, and aggregate state using a hybrid density function. The frequencies obtained from the optimized geometries were then used to confirm the stability of the configurations, and further calculations on optical properties were conducted. To investigate the dynamic process of photophysics, including fluorescence, phosphorescence, intersystem crossing, and internal conversion, we employed the MOMAP program to carry out. We also explored the effects of temperature, crystallization packing, and Duschinsky rotation on the radiative and nonradiative decay processes. This paper will provide valuable insights for future research in this field.

## Theoretical method and computational details

The fluorescence and phosphorescence radiative decay rates can be calculated using the Einstein spontaneous emission formula, which can be expressed by integrating over the whole emission spectrum:

1 $$k_{r} (T) = \int {\sigma_{emi}^{FC} (\omega )d\omega }$$

2 $$\sigma_{emi} (\omega ) = \frac{{4\omega^{3} }}{{3\hbar c^{3} }}\sum\limits_{{v_{i} ,f_{i} }} {P_{{iv_{i} }} (T)\left| {\left\langle {\theta_{{f,v_{f} }} \left| {\mu_{fi} } \right|\theta_{{i,v_{i} }} } \right\rangle } \right|^{2} \delta (\omega_{{iv_{i} fv_{i} }} - \omega )}$$

Here $\sigma_{emi}^{FC} (\omega )$ is the emission cross section with dimensions of cm^2^, *c* is the velocity of light, $P_{{iv_{i} }} (T)$ is the Boltzmann population of initial states, $\mu_{fi}$ is the electric transition dipole moment, *θ* is the vibrational wave functions, the subscript *i* and *f* refer to initial and final states, respectively, the internal conversion (IC) processes can be treated mathematically with Fermi’s golden rule by the displaced harmonic oscillator model, the IC rate can be written as follows:

3 $$k_{IC} = \sum\limits_{kl} {k_{ic,kl} }$$

4 $$k_{ic,kl} = \frac{1}{{\hbar^{2} }}R_{kl} \int_{ - \infty }^{\infty } {dt[e^{{ - i\omega_{if} t}} Z_{iv}^{ - 1} } \rho_{ic,kl} (t,T)]$$

*R*_*kl*_ is the nonadiabatic transition momentum containing both diagonal and nondiagonal, *Z*_*iv*_ is the partition function, and *ρ*_*ic,kl*_ is the thermal vibrational correlation function in the internal conversion process, which can be obtained by the multidimensional harmonic oscillator model in MOMAP package. The rate constants for intersystem crossing processes can be calculated using classical Marcus formula as follows:

5 $$k_{{\text{ISC (et)}}} = \frac{2\pi }{\hbar }\left| {H_{ij} } \right|^{2} \left( {\frac{1}{{4\pi \lambda k_{B} T}}} \right)^{1/2} \exp \left[ { - \frac{{(\Delta G^{0} + \lambda )^{2} }}{{4\lambda k_{B} T}}} \right]$$

where λ is the total reorganization energy containing inner (λ_i_) and outer reorganization energy (λ_s_) from the solvent, Δ*G*^0^ is the variation of the Gibbs free energy in the process,* k*_*B*_ is the Boltzmann constant, and T is the temperature, here it is set as 300 K. *H*_*ji*_ refers to the spin orbit coupling (SOC) and is computed by the quadratic response function method in the Dalton program^36^.

Moreover, it is possible that an excited electron in the S_1_ state can be transferred to neighboring molecules, possibly affecting the luminous mechanism and further affecting the fluorescence efficiency, this charge transfer rates (*k*_et_) for electron or hole can be theoretically calculated based on Marcus theory, according to two state model, the generalized Mulliken–Hush (GMH) and fragment charge difference (FCD) approximations are efficient and reliable to calculate charge transfer matrix elements, which depends strongly on the donor–acceptor distance and geometry of the system, the GMH can be expressed as follows^37^:

6 $$H_{ij} = \frac{{m_{ij} \Delta E_{ij} }}{{\sqrt {(\Delta \mu_{ij} )^{2} + 4(m_{ij} )^{2} } }}$$

where Δ*E*_*ij*_ is the energy gap between the initial adiabatic state and the final one, Δ*μ*_*ij*_ is the dipole moment difference between states *i* and *j*, and *m*_*ij*_ denotes the transition dipole moment connecting the two states. The FCD method to estimate the electron transfer matrix element is very similar to GMH formula as following^38^:

7 $$H_{ij} = \frac{{\Delta q_{12} \Delta E_{ij} }}{{\sqrt {(\Delta q_{1} - \Delta q_{2} )^{2} + 4\Delta q_{12}^{2} } }}$$

here, Δq_1_ and Δq_2_ are the D–A charges difference in the adiabatic states *i* and *j*, respectively, and Δq_12_ is the corresponding off–diagonal term.

Here we ignore the photochemistry reaction owing to that no chemical reaction was observed in the experiment, therefore based on the Jablonski diagram, there are four major de–excitation routes for the first singlet excited state, the first one is the radiative decay (*k*_r_) from the S_1_ to S_0_ state; the second is the internal conversion (*k*_nr_) decay from the S_1_ to S_0_ state; the third is the intersystem crossing (*k*_*isc*_) from the S_1_ to triplet T_1_ state, the last is the charge transfer (*k*_et_) from the S_1_ to the adjacent molecule, thus the fluorescence quantum yield can be expressed as *η* = *k*_r_/(*k*_r_ + *k*_nr_ + *k*_isc_ + *k*_et_), one can see the molecular light–emitting efficiency is visibly determined by the competition between the radiative decay rate (*k*_r_) and the nonradiative decay rate (*k*_nr_, *k*_isc_ and *k*_et_), according to the above equation, suppressing the non-radiative rate and increasing the radiative rate leads to a higher fluorescence efficiency, and a large intersystem crossing rate and a small anti-intersystem crossing rate are necessary if a high phosphorescence efficiency is desired.

**Figure 1:**
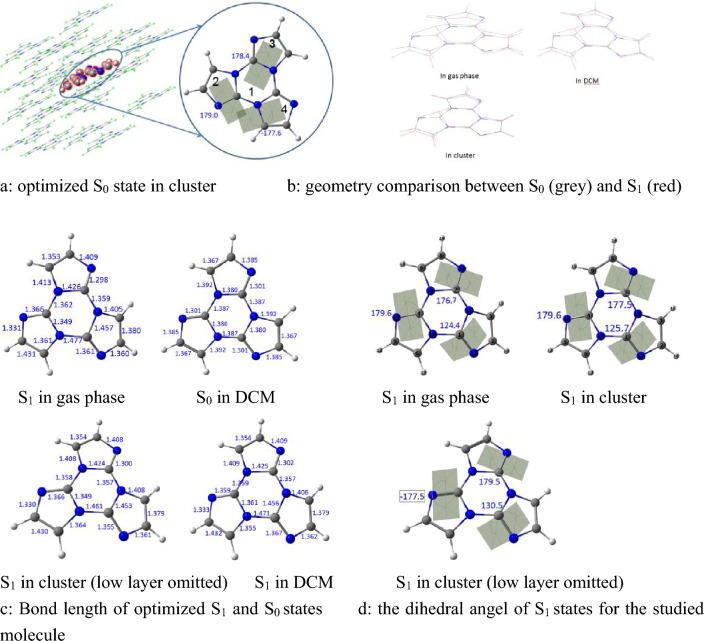
Bond parameters of the studied molecule in their minima by B3LYP/6–31G (d, p). ONIOM model (**a**): the centered molecule is treated by B3LYP/6–3131G**: UFF as a high layer and the surrounding molecules are fixed as a low layer, (**b**): geometry comparison between S_0_ (grey) and S_1_ (red), (**c**): bond length of optimized S_1_ and S_0_ states, (**d**): the dihedral angel of S_1_ states for the studied molecule.

The geometric optimization of the title compound at S_0_, S_1_ and T_1_ states were implemented using B3LYP/6–31(d, p) level in gas phase and solution, the polarizable continuum model (PCM) was employed to simulate the solvent effect on molecular photophysical properties in dichloromethane (DCM), the combined quantum mechanics and molecular mechanics (QM/MM) approach was used to model the stacking surrounding in crystal, the initial guessed structure came from the crystal cell experimentally, the ONIOM method was carried out the QM/MM calculation through Gaussian09 package. In ONIOM model (as shown in Fig. 1a), the centered molecule was calculated by quantum mechanical method as a high layer, the surrounding molecules were treated by molecular mechanics with UFF forces field as low layer, meanwhile the electronic embedding scheme was taken in this model. For the S_1_ state, the time dependent DFT was applied at the same basis set. Harmonic vibrational frequencies were calculated at the equilibrium geometries of the S_0_, T_1_ and S_1_. In addition, based on the electronic structure of the title compound, the Huang–Rhys factor, normal mode displacements, nonadiabatic electronic coupling between the two electronic states were implemented using MOMAP, at last the radiative and nonradiative decay rates were computed and the fluorescent quantum yield was obtained also.

## Results and discussion

### The geometry and normal modes analysis

**Figure 2:**
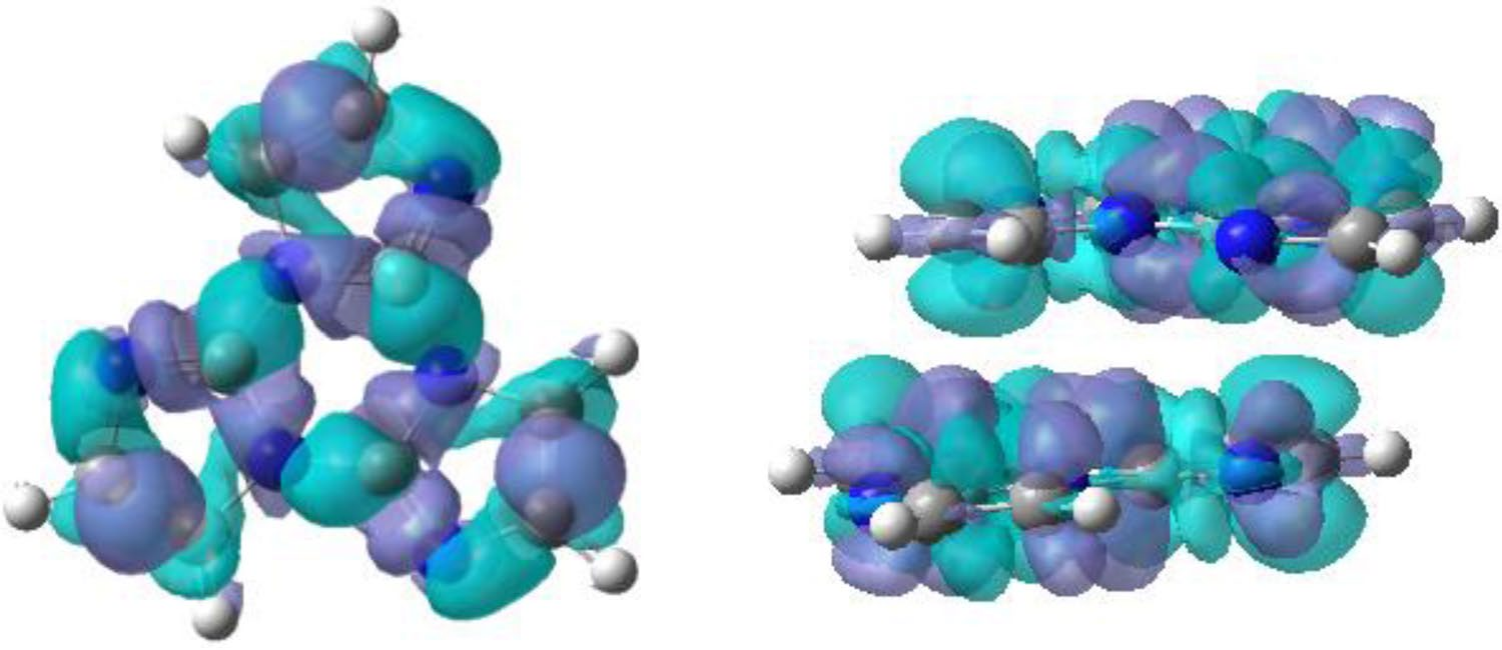
Electron density difference map between S_0_ and S_1_ in DCM (left) and in dimer (right), note the loss of electron density at the blue area and the gain in electron density at the purple area corresponding to S_0_ → S_1_.

Our optimized S_0_ minima of the title compound is a exactly planar molecule with *C*_*3h*_ symmetry in gas phase and in DCM, however, this molecule in aggregate state seems to be not planar and its dipole moment is 0.2271 Debye, Fig. 1a shows the dihedral angles of three imidazole rings (marked as 2, 3 and 4) and triazine ring 1 are 179.0°, 178.4° and – 177.6° respectively, which means the central molecule is slightly distorted due to the interaction from the adjacent surrounding. Nevertheless, all the optimized excited states S_1_ and T_1_, whether in gas phase, in DCM or in cluster, become nonplanar, it is found that the triazine ring 1 at S_1_ states is completely twisted, but each imidazole ring (refer to ring 2, 3 and 4) remains basically on the same plane, it should be pointed out that the title compound consisting of three imidazole rings is nonplanar. Figure 1b visually illuminates the changes of the S_0_ → S_1_ minima in different surroundings, the extent of variation for molecule-in-cluster is the smallest, and that in vacuum is the largest, though their variational tendency is the same. The three dihedral angles of the S_1_ minima in gas phase, DCM and cluster are shown in Fig. 1d, in which the maximum deviations from plane are 124.4°, 125.7° and 130.5° correspond to gas phase/DCM/cluster, respectively, these data again confirm quantificationally the changing trendy in Fig. 1b. The three C–N bond in triazine ring for S_1_ minima, which do not share with the imidazole rings, for the isolated molecule are 1.359, 1.362 and 1.477 Å, for the solvated molecule are 1.357, 1.359 and 1.471 Å, as for the locked molecule in cluster, those become 1.357, 1.358 and 1.461 Å (shown in Fig. 1c, the data of S_0_ in vacuum and cluster attached in Supplementary Information), respectively, we can see these bond parameters in cluster have a minimum amount of change compared with these in vacuum and solution, it is clear that the intermolecular interactions constrain the deformation of the molecular geometry. In addition, it should be pointed out that the planar S_0_ minima show obvious conjugative characteristic, here all the C–C and C–N bonds at the S_0_ minima are actually shorter than typical single bonds, but longer than typical double bonds, for example, Fig. 1c shows all the C–C bonds are 1.367 Å and C–N bonds are between 1.301 and 1.392 Å at S_0_ minimum in DCM, however, the three C–C bonds in S_1_ minimum are unequal owing to broken symmetry, one shortens to 1.356 Å, while the other two lengthen to 1.379 and 1.432 Å, as for C–N bonds in S_1_ state, which in three imidazole rings seem irregularity to follow, they change between 1.302 and 1.471 Å and possess typical characteristic of single bond and double band, which illustrates the π–conjugated system is completely destroyed, the electron density-difference map (Fig. 2) between the S_0_ and S_1_ states may explain the change, we can find the electron shift from blue area with negative value to purple area with positive value, that is, the C–N bonds corresponding to electron shifting out will be lengthened, while the C–N bonds corresponding to electron entering into will be shortened. Similarly, this situation also exists in the T_1_ state in gas phase, solution and cluster, referring to Supplementary Information for details (Figs. S1 and S2).

**Figure 3:**
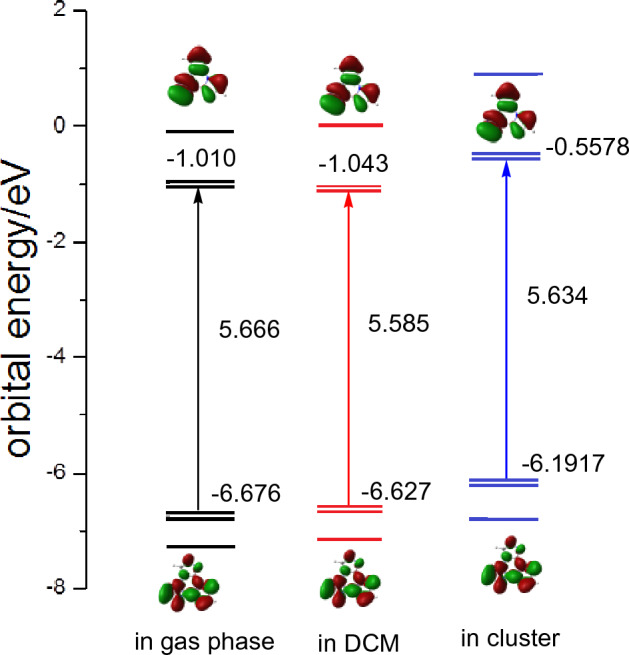
Energy levels diagrams of diimidazole molecule calculated based on the optimized S_0_ state in gas phase, DCM and cluster, the molecular orbitals are shown together (isovalue = 0.02).

Furthermore, we investigated the effect of intermolecular interaction on frontier molecular orbital, the calculated energy level, energy gap between frontier molecular orbital and the corresponding distributions of the highest occupied molecular orbital (HOMO) and the lowest unoccupied molecular orbital (LUMO) are shown in Fig. 3 and Fig. S3. One can find that both the HOMO and LUMO maps in three environments have hardly change and indicate π characteristic, but the HOMO is a bonding orbital and LUMO is an anti–bonding orbital, in addition, owing to the molecular symmetry of S_0_ minima, all the HOMOs and LUMOs are double degenerate. While there are significant changes in the frontier orbital energies, it can be observed that the HOMO energy levels increase successively in the gas phase (– 6.676 eV), solution (– 6.627 eV), and cluster (– 6.192 eV). It is worth noting that the LUMO energy level in DCM is lower than that in the gas phase, while in the cluster, it increases. Additionally, as for the energy gap between HOMO and LUMO, the smallest value of 5.585 eV is observed in DCM, followed by the cluster with 5.634 eV, and the maximum value of 5.666 eV in the gas phase.

### Environment effect on photophysical properties, Huang–Rhys factor and reorganization energy

The photophysical properties for S_1_ state of the title compound, including the vertical excitation energy, oscillator strength (*f*), main configuration and the assignment, are compiled and listed in Table 1. During the absorption process, the vertical excitation energies keep nearly constant under different conditions (230 nm), which matches well with the experimental observations. The main transition configuration undergoes slight changes between the solution and solid state, besides HOMO–1 → LUMO and HOMO → LUMO + 1 in DCM and in cluster, for solution HOMO–2 → LUMO + 1 is one of the main transition configuration, and for cluster HOMO–2 → LUMO is main transition also, while the oscillator strength is smaller slightly in solid phase (0.1493) than that in DCM (0.1634). Our investigation of the emission process revealed that the transition configuration from HOMO to LUMO was the dominant process in both the DCM and solid phases. Nevertheless, we observed a significant decrease in oscillator strengths compared to the excitation process. Additionally, it was discovered theoretically that the maximum emission wavelength was redshifted from solid phase (388.6 nm) to DCM (405.6 nm). These theoretical predictions were in good agreement with experimental data (390 nm (10^–4^ M), 400 nm (10^–2^ M) in DCM and 400 nm in crystal, 425 nm in powder), where the maximum emission peaks were observed to be 400 nm in the crystal and 425 nm in the powder. In addition, we studied some dynamic parameters about photophysics, in which the radiative and nonradiative rates, the ISC and RISC rates, and the rate of photoinduced electron transfer were involved. It is well–known Huang–Rhys factor (HR) and reorganization energy (λ) are crucial parameters to estimate these rates with formula 2, 4 and 5^39,40^, thus both the values have been obtained firstly using MOMAP package and part are shown in Table 2 (all the data are shown in Tables S1, S2, S3 and S4), the associated nonadiabatic coupling term R, the corresponding normal vibrational frequencies and the Franck–Condon (FC) factors are listed together.

**Table 1:** Absorption and emissions of the title compound according to TDDFT calculations, together with the experimental data.

Environment	Oscillator strength	Main configuration	Assignment	λ_cal_ (nm)	λ_exp_ (nm)
The predicted absorption data of the title molecule					
In gas phase	0.1700	HOMO → LUMO	0.4111	230.1	
		HOMO–1 → LUMO + 1	0.4117		
		HOMO–2 → LUMO + 1	– 0.3086		
In DCM	0.1634	HOMO–1 → LUMO	0.4192	230.1	230
		HOMO → LUMO + 1	0.4188		
		HOMO–2 → LUMO + 1	0.3234		
In cluster	0.1493	HOMO–1 → LUMO	0.3560	232.1	
		HOMO–2 → LUMO	– 0.3405		
		HOMO → LUMO + 1	0.3351		
The predicted emission data of the title molecule					
In gas phase	0.0371	HOMO → LUMO	–0.7014	421.1	
In DCM	0.0851	HOMO → LUMO	–0.7032	405.6	390 (10^–4^ M), 400 (10^–2^ M)
In cluster	0.0386	HOMO → LUMO	0.7004	388.6	400 (crystal), 425 (powder)

B3LYP/6–31g (d, p):uff = qeq method is employed in cluster.

**Table 2:** The selected reorganization energy (λ_reorg_/cm^–1^) by NMA method in S_1_ → S_0_ process in cluster, frequencies of S_1_ state, nonadiabatic coupling term (R), Huang–Rhys factor (HR), and Franck–Condon factors (FC).

	Freq. (cm^–1^)	R (cm^–1^)	λ_reorg_ (cm^–1^)	HR	FC
In cluster					
v_2_	143.6	225.6	193.2	1.3452	0.0215
v_5_	259.2	10.7	103.4	0.3988	0.0096
v_6_	284.9	43.9	151.8	0.5327	0.0153
v_7_	310.5	201.6	459.9	1.4812	0.0191
v_9_	441.5	62.3	81.7	0.1851	0.0018
v_11_	515.1	825	428	0.8309	0.0237
v_13_	561.9	95.2	1129.8	2.0104	0.0098
v_14_	577.9	32.8	256.9	0.4445	0.0116
v_33_	1105.2	94.5	445.6	0.4032	0.0098
v_38_	1221.1	27.3	556.4	0.4556	0.0121
v_46_	1478.5	2487.5	947.9	0.641	0.0192
v_50_	1661	31.1	1027.1	0.6183	0.0185
v_51_	1712.3	149.5	1274.7	0.7444	0.0221
In solvent					
v_3_	111.1	8.4	1622.6	14.6008	0
v_16_	633.2	1.6	2178.1	3.4395	0.0013
v_51_	1687	10.1	1110.3	0.6579	0.0396

**Figure 4:**
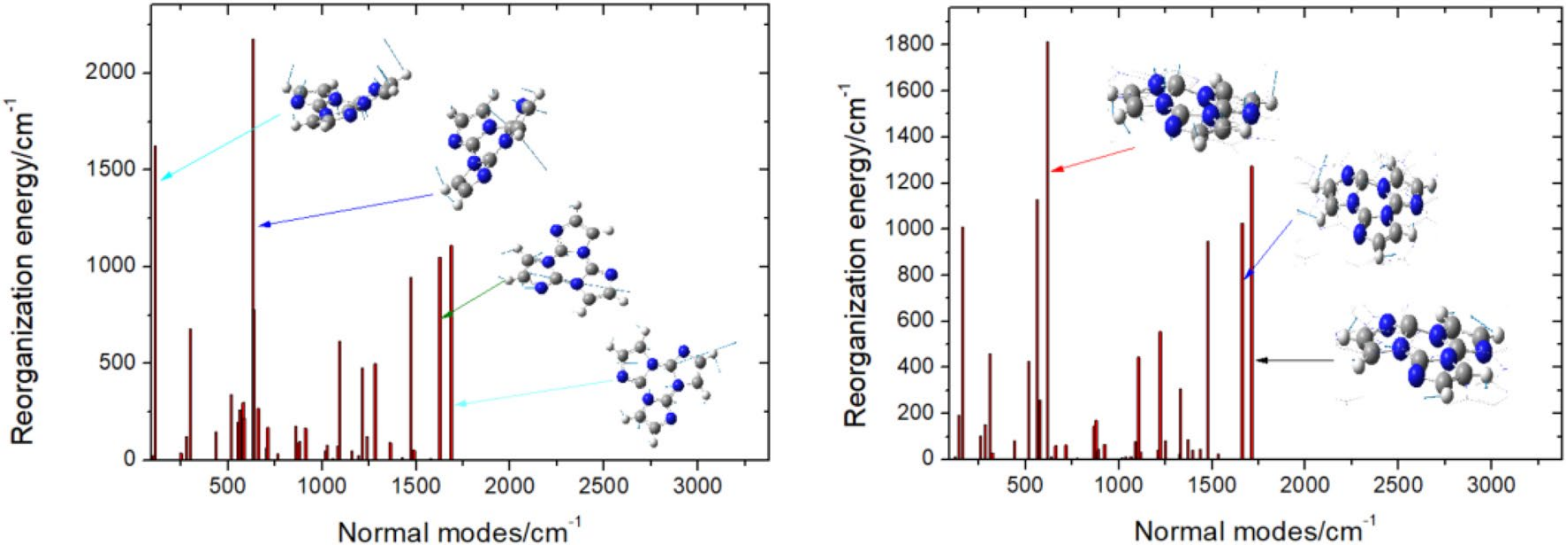
Calculated the reorganization energies from the normal modes in DCM and in cluster.

When considering the fluorescence process, we discovered that the total reorganization energy in the cluster (11,359 cm^–1^, or 1.408 eV) is smaller than that in DCM (13,467 cm^–1^, or 1.670 eV), which was consistent with the aforementioned geometrical changes. Using the normal mode analysis (NMA) method^41,42^, we can conclude that in a solution environment, the vibrational modes with 1687, 633.2, and 111.1 cm^–1^ frequencies present the largest reorganization energies after analyzing the data, however, when considering the modes in a cluster, their frequencies become 1712, 1661, and 561.9 cm^–1^ respectively (see Tables S1, S3, S4 and Fig. 4). The insets in Fig. 4 visually depict that the main contribution to reorganization energies comes from low–frequency and middle–frequency modes. It is evident from the data that the λ values in DCM are generally larger than those in the cluster due to the molecular stacking effect.

**Figure 5:**
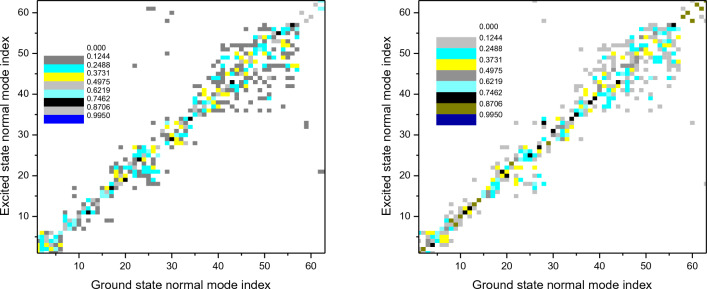
Duschinsky rotation matrix of triimidazole molecule corresponding to S_1_ → S_0_ process in DCM and in cluster.

Because reorganization energy is proportional to Huang–Rhys factor ($\lambda_{{\text{i}}} = hvHR_{i}$), and accordingly, the larger the λ is, usually the larger the HR is. It can be seen that the largest HR for the title compound is 2.010 in cluster, which is associated with the vibrational mode of frequency 561.9 cm^–1^, and is assigned to the twisted vibration of triazine rings as shown in Fig. 5, both the other vibrational modes with low frequencies (143.6 and 310.5 cm^–1^) possess the second large HR values (corresponding 1.3452 and 1.4812), they correspond to the twisted vibration of triazine rings as well, furthermore, middle–frequency vibrations (1478.5, 1661, and 1712.3 cm^–1^) make a significant contribution to HR (0.641, 0.6183, and 0.7444) too, which are assigned to the C–C and C–N stretching vibration in triazine rings.

To probe the mechanism of the emissive behavior about the studied system, we investigated the lifetime of the excited state and fluorescence quantum yield of the title compound in DCM and cluster, the calculated *k*_r_ and *k*_nr_ are listed in Table 3. It can be found that: (i) taking Duschinsky rotation effect (DRE) into account, the calculated *k*_r_ of the title compound are 1.617 × 10^7^ s^–1^ in DCM and 1.862 × 10^7^ s^–1^ in cluster at 300 K, the corresponding fluorescence lifetime are 61.8 ns and 53.7 ns respectively, unfortunately the observed in cluster is 15.29 ns and in DCM is 7.84 ns, obviously, there remains a considerable gap between the theoretical and calculated values, but their trend of change is consistent; (ii) no matter in DCM or in cluster, the predicted *k*_nr_ are almost 3-orders larger than the *k*_r_ under the same conditions, if ignoring DRE, the *k*_r_ and k_nr_ decrease significantly to 10^4^ s^–1^ and 10^6^ s^–1^ in DCM; (iii) when the temperature goes up from 50 to 300 K, the *k*_r_ and *k*_nr_ in cluster changes very little compared to the vacuum and solution conditions.

**Table 3:** Calculated radiative decay rate (*k*_r_) and nonradiative decay rate (*k*_nr_) from S_1_ to S_0_ and the corresponding fluorescence quantum yield (*η*) at different temperatures for the title molecule.

Duschinsky rotation effect (DRE)				No Duschinsky rotation effect (DRE)		
T (K)	*k*_r_ (s^–1^)	*k*_nr_ (s^–1^)	*η*	*k*_r_ (s^–1^)	*k*_nr_ (s^–1^)	*η*
In vacuum						
50	2.342 × 10^7^	4.775 × 10^11^		8.000 × 10^3^	1.124 × 10^7^	
100	2.221 × 10^7^	4.457 × 10^11^		1.027 × 10^4^	1.255 × 10^7^	
150	2.220 × 10^7^	4.775 × 10^11^		1.027 × 10^4^	1.471 × 10^7^	
200	2.152 × 10^7^	3.856 × 10^11^		1.250 × 10^4^	1.804 × 10^7^	
250	2.083 × 10^7^	3.621 × 10^11^		1.574 × 10^4^	2.308 × 10^7^	
300	2.015 × 10^7^	3.425 × 10^11^		2.030 × 10^4^	3.064 × 10^7^	
In DCM						
50	7.053 × 10^7^	9.137 × 10^10^		6.014 × 10^3^	5.357 × 10^5^	
100	7.014 × 10^7^	1.084 × 10^11^		6.579 × 10^3^	5.938 × 10^5^	
150	6.942 × 10^7^	1.285 × 10^11^		7.596 × 10^3^	6.945 × 10^5^	
200	6.856 × 10^7^	1.477 × 10^11^		9.177 × 10^3^	8.572 × 10^5^	
250	6.761 × 10^7^	1.653 × 10^11^		1.150 × 10^4^	1.114 × 10^6^	
300	1.617 × 10^7^	1.811 × 10^11^	8.93 × 10^–3^%	1.483 × 10^4^	1.514 × 10^6^	0.98%
In cluster						
50	2.057 × 10^7^	1.149 × 10^10^		1.302 × 10^7^	7.191 × 10^7^	
100	2.033 × 10^7^	1.337 × 10^10^		1.299 × 10^7^	7.437 × 10^7^	
150	1.997 × 10^7^	1.684 × 10^10^		1.294 × 10^7^	8.004 × 10^7^	
200	1.955 × 10^7^	2.196 × 10^10^		1.288 × 10^7^	8.983 × 10^7^	
250	1.909 × 10^7^	2.885 × 10^10^		1.281 × 10^7^	1.048 × 10^8^	
300	1.862 × 10^7^	3.765 × 10^10^	0.049%	1.280 × 10^7^	1.264 × 10^8^	9.19%

The calculated phosphorescence radiative rates *k*_p_ in vacuum/solution/cluster are 0.126/0.128/0.130 s^–1^ at 300 K, respectively. The experimental data of the fluorescence quantum yield is 2% in DCM, and 18% in solid state^31^.

In general, the non–radiative decay process will dissipate the more excited energy when the reorganization energy in this process is larger^16^, considering Duschinsky rotation and mode distortion, the twisting motion with low–frequency and the stretching motion with middle–frequency in DCM couple strongly with the electronic excitation, which dissipates the energy efficiently by a fast decay rate (5.52 ps, the inverse of *k*_nr_), while the restricted molecule in cluster, these motions are impeded by the intermolecular forces with a much slower decay rate (26.6 ps). The caused mode mixing by the Duschinsky rotation is graphically shown in Fig. 5 and it can be seen the modes with low- and middle-frequency play an important role^43^, we found the mixing of low-frequency mode in solution is more serious than that in cluster. In addition, because the title molecule in cluster is surrounded by adjacent molecules, the proportion of the displaced harmonic oscillator increases while that of the distorted displaced harmonic oscillator decreases, it can be inferred that the Duschinsky effect during the emission process is weakened^44,45^, therefore the *η* value will increase theoretically, if we solely took into account the radiation decay and internal conversion without Duschinsky effect, the fluorescence quantum yield (9.19%) of the triazinethe in cluster is 9.4 times higher than that (0.98%) in DCM, when Duschinsky effect is under consideration, the *η* value is expected to increase 55-fold from 8.93 × 10^–3^% in DCM to 0.049% in cluster. According to the above theoretical analysis, we believed that the low-frequency and middle-frequency motions in solid phase are hindered and the energy dissipation pathway by the internal conversion are slowed down, thus the AIE characteristic of the title molecule from DCM to solid phase is found.

Moreover we computed the approximate FC factors of all the normal modes to simulate the vibronic fluorescent spectrum of the title molecule in cluster. Based on the harmonic oscillator model, the FC factor can be expressed by a Poisson distribution as follows^46^:

6 $$\mathop \prod \limits_{k} \left| {\left\langle {\theta_{{fv_{k} }} |\theta_{i0} } \right\rangle } \right|^{2} = \mathop \prod \limits_{k} \frac{{S_{k}^{{v_{k} }} }}{{v_{k} !}}e^{{ - S_{k} }}$$

**Figure 6:**
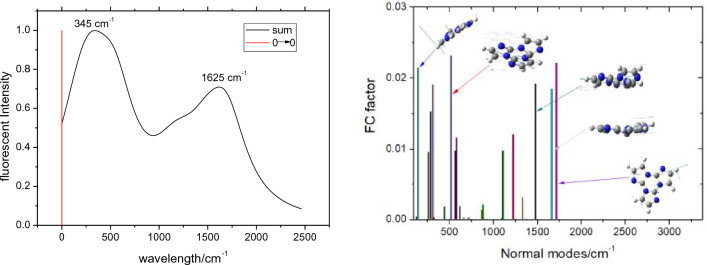
Simulated fluorescence spectra of triimidazole molecule using the discrete spectral line method (left) and Franck–Condon factor analysis vs normal modes (right).

For both the different electronic states of a molecule in aggregate state, a small displacement in the potential energy surface can be anticipated due to the steric inhibition, and the harmonic oscillator model should be a good approximation. Here for simplicity, we only considered the contribution to the spectrum of the 0 → 0, 0 → 1 and 0 → 2 vibrational transitions, the other higher vibrational transitions were neglected, thereby the FC factor about vibrational modes will be simplified as $\frac{1}{2}S_{{\text{k}}}^{3} e^{{ - 3S_{{\text{k}}} }}$, S_k_ is the HR factor of the *k*th mode, some selected modes are listed in Table 4 (other shown in Table S4), it can be found that the FC factors with low frequencies 143.6, 310.5, 515.1 cm^–1^ are fairly large, which impact signally on the shape of the emission spectrum, we adopted Lorentzian function to fit the emission spectrum with 500 cm^–1^ full width at half maximum (FWHM), the spreading linetype of the modes with low–frequency will overlap together with the main peak (referring to 0 → 0 electron transition), which makes the main emission peak red shift about 345 cm^–1^, i.e. corresponding 394 nm. The modes with 1478.5, 1661.0, and 1712.3 cm^–1^ also make a significant contribution to HR and the spectrum, it leads to the shoulder peak in emitting spectrum and the peak position is 1625 cm^–1^, i.e., corresponding 415 nm, which exactly matches with the shape of the emitting spectrum as detected in experiment (main peak is 400 nm and shoulder peak is 420 nm). Figure 6 diagrammatized the simulated vibronic emissive spectrum and the distribution characteristics of FC factors. Additionally Fig. S4 presented the simulated emissive spectra of the studied molecule with FWHM 300 and 400 cm^–1^ as a contrast.

**Table 4:** Spin–orbital coupling (SOC)/cm^–1^, reorganization energy (λ_reorg_)/eV between the S_1_ and T_1_ states using NMA and the DPES methods, adiabatic energy difference ΔE of both the S_1_ and T_1_ states, intersystem crossing rate (*k*_isc_), and reverse intersystem crossing rate (*k*_risc_) of the title compound at the S_1_ and T_1_ minima.

	In gas phase		In DCM		In cluster	
	S_1_ → T_1_	T_1_ → S_1_	S_1_ → T_1_	T_1_ → S_1_	S_1_ → T_1_	T_1_ → S_1_
SOC (cm^–1^)	4.19	3.68	4.01	3.70	2.98	2.23
λ_reorg_ )eV)	3.408	2.573	2.094	2.107	2.349	2.029
ΔE (eV)	–4.395	4.395	–5.413	5.413	–0.879	0.879
*k*_isc_ or *k*_risc_ (s^–1^)	3.262 × 10^6^	2.091 × 10^–8^	2.340 × 10^6^	4.793 × 10^–10^	2.041 × 10^5^	2.912 × 10^–9^

The λ_reorg_ is from NMA method with internal coordinate, the temperature is 300 K, *k*_isc_ corresponds to S_1_ → T_1_ and *k*_risc_ corresponds to T_1_ → S_1_.

### ISC, RISC and photo induced charge transfer rates

Experimentally, fluorescence and phosphorescence of the title molecule were detected simultaneously. As is well established, the process of phosphorescent emission is closely related to intersystem crossing, to gain insight into this photophysical mechanism, we used the classical Marcus theory to investigate the intersystem crossing rate (*k*_isc_) and the reverse intersystem crossing rate (*k*_risc_). Table 4 provided information on the adiabatic energy difference ΔE_S1–T1_ between the S_1_ and T_1_ states, as well as the reorganization energies (λ_reorg_) and spin-orbital coupling (SOC). Here the λ_reorg_ were from NMA with internal coordinate^47^, SOC were carried out using Dalton program.

It can be found that the ΔE_S1–T1_ is particularly sensitive to the environment, when the title molecule is put into DCM, the computed ΔE_S1–T1_ is – 5.413 eV, when the molecule is locked in cluster, the ΔE_S1–T1_ decreases dramatically to – 0.879 eV. In comparison, the associated λ_reorg_ in solution and crystal are not changing much, of which the S_1_ → T_1_ process is 2.094 eV in DCM and the reverse process T_1_ → S_1_ is 2.107 eV. In cluster, the λ_reorg_ of ISC process becomes 2.349 eV and the reverse process decreases to 2.029 eV. For details about reorganization energy using NMA method, each value vs vibrational mode is given in Table S4.

Because of lack of the relativistic effect from heavy atom in the title compound, as predicted, the SOC data are considerably small and less than 5 cm^–1^, when temperature is set as 300 K and *H*_ij_ takes SOC value, the estimated *k*_isc_ and *k*_risc_ are obtained according formula 5 and compiled in Table 4. It can be found the *k*_isc_ is 2.340 × 10^6^/2.041 × 10^5^ s^–1^ in DCM/cluster, for reverse process, the *k*_risc_ is 4.793 × 10^–10^/2.912 × 10^–9^ s^–1^ in DCM/cluster. Compared with the radiative rate *k*_isc_, we observed the *k*_risc_ in DCM or in cluster are very smaller, even can be completely ignored, thus it seems that the intersystem crossing process is remarkable but the reverse intersystem crossing is impossible, accordingly the delayed fluorescence from RISC process is improbable too. We have obtained the phosphorescent emission rates from the T_1_ to S_0_ state theoretically using the Dalton program. In DCM, the rate is 0.128 s^–1^ and in the cluster, the rate becomes 0.130 s^–1^. These rates are higher than the *k*_risc_ values, suggesting that experimental observation of the phosphorescent phenomenon is likely. The corresponding lifetime of the phosphorescence in DCM/cluster was determined to be 7.81 s/7.69 s, respectively, which is similar to the measured values of 0.969 s and 0.99 s. The predicted phosphorescent emission peaks based on the vertical excited energy between the S_0_ and T_1_ minima are 640.8 nm in DCM (500 nm measured) and 536.8 nm in the cluster (525 nm measured). These calculated results indicated that the theoretical value in solid state was in accordance with the experimental one in the phosphorescent emission process, but one in DCM is far from the observed value, which could be attributed to polarization continuum model, this model may not fully capture the intricate interactions between the solute and solvent molecules, leading to inaccuracies in the calculated spectra. In addition, the choice of the exchange–correlation functional within DFT can affect the accuracy of the calculations, certain functionals may not properly account for the effects of excited-state and solvent–solute interactions, leading to deviations from experimental results (Table 5).

**Table 5:** Distance of dimer, λ_reorg_ and charge transfer coupling with GMH and FCD approximations corresponding to excited charge transfer process, and charge transfer rate (*k*_et_).

	Distance (Å)	λ_reorg_ (eV)	GMH (eV)	ΔE (eV)	*k*_et_ (s^–1^)	FCD (eV)	ΔE (eV)	*k*_et_ (s^–1^)
Electron transfer (ωB97X/6–31G (d,p))								
Path 1	3.72	1.05	0.157	0.315	1.42 × 10^5^	0.157	0.315	1.42 × 10^5^
Path 2	3.96	1.05	0.072	0.144	1.68 × 10^5^	0.072	0.144	1.68 × 10^5^

**Figure 7:**
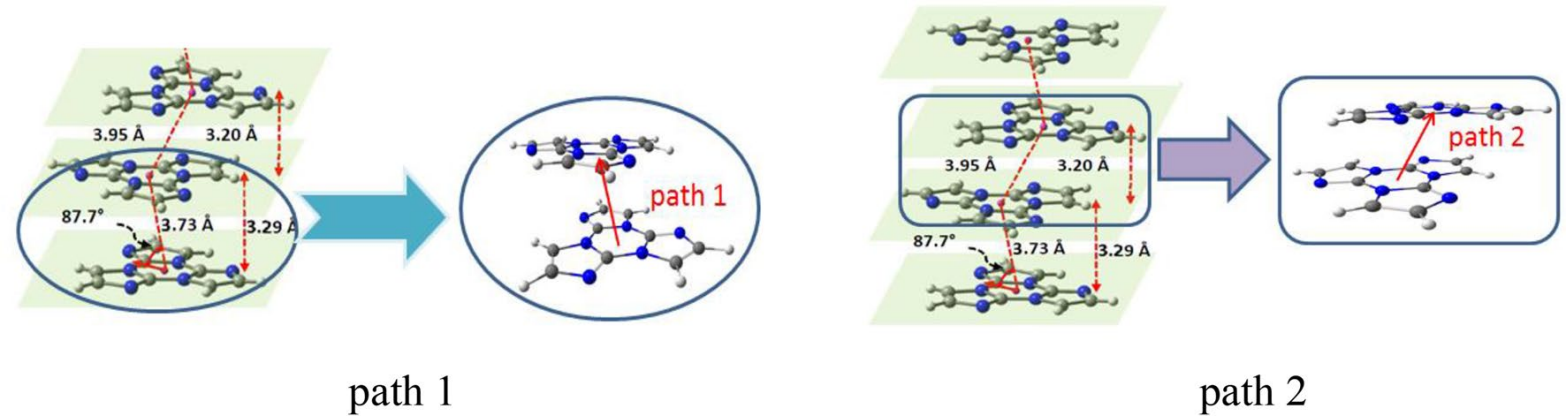
The intermolecular distances for path 1 and path 2 in dimers.

In the S_1_ state of the title compound, the excited electron has the possibility of transferring to adjacent molecules—so called photo-induced intermolecular electron-transfer^48–50^, there are two paths for electron transfer at random and shown in Fig. 7 (path 1 and path 2 for short). With the aid of the crystal structure, we can find the inter-centroids separation is 3.73 Å for the dimer of path 1 and 3.95 Å for path 2, but the perpendicular distance between both the molecular planes for path 1 is 3.29 and path 2 is 3.20 Å. When an excited electron in the donor goes to a neighboring molecule, the excited molecule will quickly relax to the ground state with a positive charge, meanwhile the acceptor with a negative charge will reorganize to its lowest energy state too, thus the reorganization energy includes two parts, which are associated with change of molecular geometries above mentioned, according to ONIOM model, the λ_reorg_ is 1.05 eV by the displacement of potential energy surface method in this case. When involved both dimers were taken out from its unit cell, only wavefunction and energy consistence were done and no geometrical optimization performed, the electronic coupling strength (*H*_ab_ instead of *H*_ij_ to distinguish) can be calculated using GMH and FCD approximations by ωB97X/6–31G(d,p), the *H*_ab_ of path 1 is 0.157 eV with an open source software^51^, however, the *H*_ab_ of path 2 decreases to 0.072 eV. Here it is important to emphasize we can observe that the electron density and its difference are both distributed on both molecules simultaneously from the dimer in Fig. 2, this suggests that due to the presence of strong intermolecular interactions, the characteristics of electron transfer seem less pronounced when the molecule transitions from the ground state to the excited state. At the same time, we calculated the hole-electron Coulomb attractive energy (exciton binding energy) in dimer which is 4.12 eV by Multiwfn program^52^, this estimated value indicates that charge transfer in the dimer is not easy^29^.

From a quantum mechanics perspective, *H*_ab_ is proportional to the orbital overlap of the wave function between the donor and acceptor during the electron transfer process. By examining the molecular configuration overlap in the dimer of path 1 and path 2 in Fig. 6, it can be concluded that the overlap between the monomers in path 1 is greater, resulting in a larger *H*_ab_ than path 2. Nevertheless, the plane spacing between both the molecular planes for the path 1 is larger than one of the path 2, which will decrease the electron transfer rates, as the energy difference ΔE of the donor and acceptor instead of the driving force, the photo–induced electron transfer rates *k*_et_ for path 1 is 1.42 × 10^5^ s^–1^ according to Marcus theory, while the *k*_et_ for path 2 is 1.68 × 10^5^ s^–1^.

Up to now, we have obtained the radiation decay rate (*k*_r_), internal conversion rate (*k*_nr_), and intersystem crossing rate (*k*_isc_) of the title compound in DCM and in cluster, in addition, the electron transfer rate (*k*_et_) in cluster was gained as well. According to the formula *η* = *k*_r_/(*k*_r_ + *k*_nr_ + *k*_isc_ + *k*_et_), the simulated fluorescence quantum yield of this material is 8.93 × 10^–3^% in DCM, and 0.049% in cluster under the DRE, when the DRE was ignored, the *η* increases to 0.98% in DCM (here no electron transfer) and 9.19% in cluster with the effect of electron transfer, here the calculated *k*_nr_ is extremely large compared with the other rates, which determines the simulated *η* of the material decisively, it has been discovered that the fluorescence quantum yield is not significantly affected by the absence of electron transfer and intersystem crossing.

## Conclusion

In summary, employing QM/MM method and Marcus theory, we explored theoretically the photophysical and charge transfer properties of cyclic triimidazole (C_9_H_6_N_6_). Results showed that, deformation of out–plane of triazine ring and imidazole motions are responsible for the photophysical properties, and triazine ring’s deformation and imidazole motions are effectively suppressed in solid phase. Therefore, HR factors and reorganization energies are smaller in aggregate state in comparison with those in DCM, and energy consumption pathways for the internal conversion from excited state to ground state are hindered, this brings the AIE characteristic of the title compound, the calculated fluorescence quantum yield *η* is 0.98% in DCM and 9.19% in cluster without DRE respectively. The simulated emission spectrum by discrete spectral lines indicated the main peak is affected by the lower-frequency modes and shoulder peak of the emission spectrum is affected by the modes with middle frequency. Furthermore, the predicted *k*_isc_ and *k*_risc_ about ISC and RISC process using Marcus theory confirm that the electron can successfully shift from the S_1_ to T_1_ state, however, the reverse T_1_ → S_1_ process cannot come into being duo to very small *k*_risc_ (10^–6^–10^–9^ s^–1^), so the phosphorescence can be observed. At last, we explored the mechanism about the electron transfer from the excited state S_1_ to the adjacent molecule, our theoretical data declared this process can be ignored due to its low electron transfer rate.

## Supplementary Information

Supplementary Information.

## Data Availability

The data that support the findings of this study are available from the corresponding author upon reasonable request.
