# Supplementary material for: Understanding H-aggregates crystallization induced emissive behavior: insights from theory
Source: Sci Rep. 2023 Jul 31;13:12357. doi: 10.1038/s41598-023-39605-5 (PMC10390577; doi:10.1038/s41598-023-39605-5)
Supplement: Supplementary file 1 — Supplementary Information. [file 41598_2023_39605_MOESM1_ESM.docx]

***Supplementary Information for***

**Understanding H–aggregates Crystallization Induced Emissive Behavior：Insights from Theory**

Huixue Li*, Kun Yuan, Lingling Lv, Sujuan Pane, Zhifeng Li*

School of Chemical Engineering and Technology, Tianshui Normal University, Tianshui, Gansu 741001, China

**Table of contents**

page

**Figure S1.** Bond length of the studied molecule for optimized T_1_ states in gas phase/DCM/cluster by QM/MM. S2

**Figure S2**. Dihedral angles of T_1_ states for the studied molecule S2

**Figure S3**. Frontier molecular orbital diagrams of the studied molecule S3

**Figure S4**. Photoluminescence and phosphorescence of the crystals at 298 K S3

**Table S1.** The frequencies of the title compound according to QM and ONIOM calculations S4

**Table S2**. The reorganization energy (λ_reorg_/cm^–1^) and Huang–Rhys factor (HR) between S_1_ and T_1_ minima by NMA method with internal coordinate S6

**Table S3.** The reorganization energy (λ_reorg_/cm^–1^), Huang–Rhys factor (HR) and nonadiabatic coupling matrix elecment between S_0_ and S_1_ minima of the title compound by NMA method with internal coordinate in gas phase and in DCM S8

**Table S4.** The reorganization energy (λ_reorg_ /cm^–1^), Huang–Rhys factor (HR), nonadiabatic coupling matrix elecment and Franck–Condon factor between S_0_ and S_1_ minima of the title compound by NMA method with internal coordinate in cluster S10


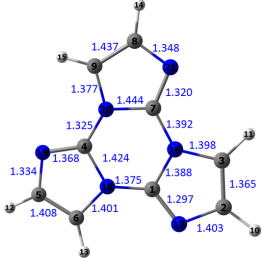

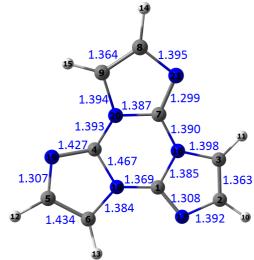


a: optimized T_1_ state in gas phase b: optimized T_1_ state in DCM


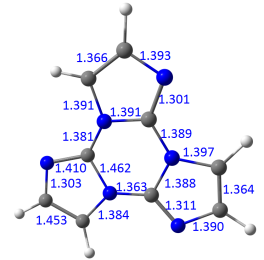

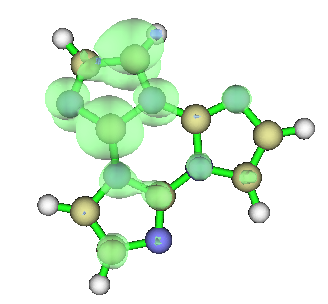


c: optimized T_1_ state in cluster d: spin density map of T_1_ state in DCM


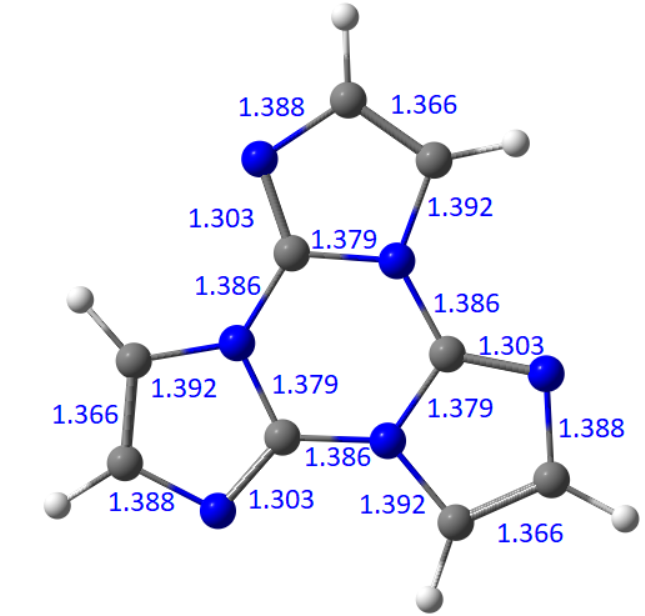

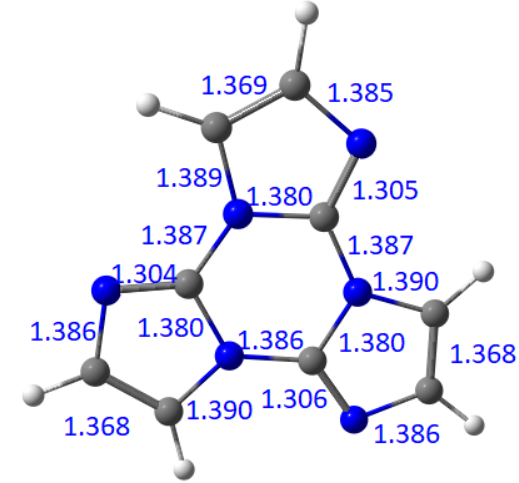


e: S_0_ in gas phase f: S_0_ in cluster

**Figure S1.** All bond lengths of S_0_ and T_1_ states for the studied molecule by B3LYP/6–31G(d, p).

ONIOM model (c): the centered molecule is treated as a high layer and the surrounding molecules are fixed as a low layer. ONIOM (B3LYP/6–31G(d):UFF), the low layer omitted.


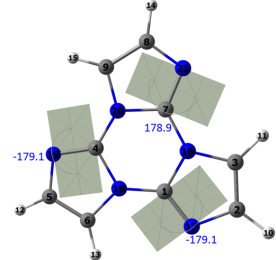

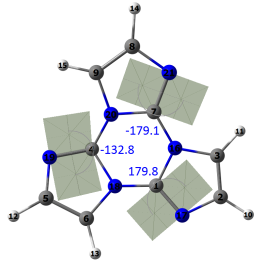


a: optimized T_1_ state in gas phase b: optimized T_1_ state in DCM


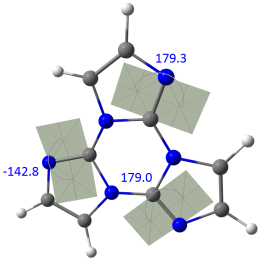


c: optimized T_1_ state in cluster

**Figure S2**. dihedral angles of T_1_ states for the studied molecule


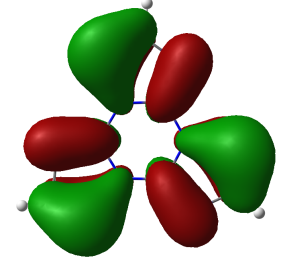

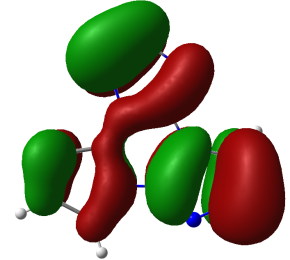

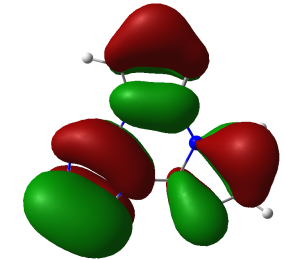


HOMO–2 HOMO–1 HOMO


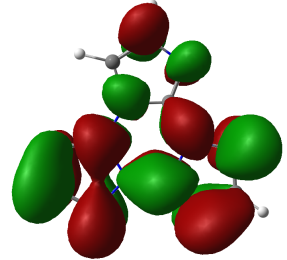

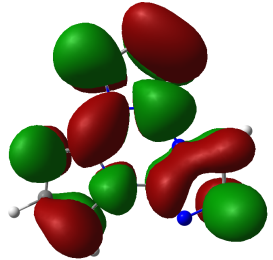

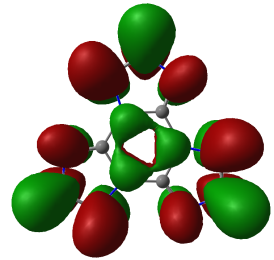


LUMO LUMO +1 LUMO +2

**Figure S3.** Frontier molecular orbital diagrams of the studied molecule


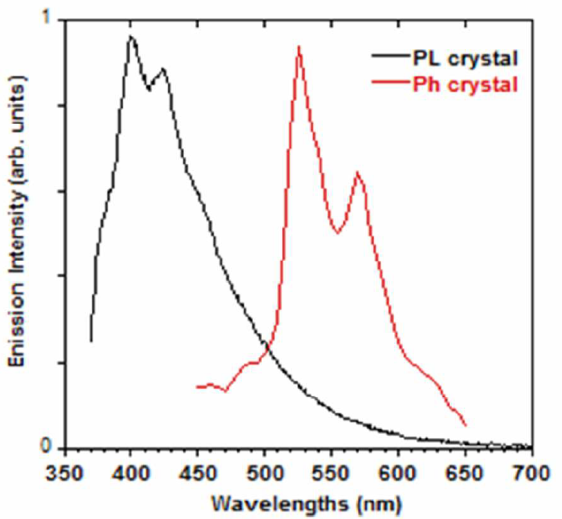


the Photoluminescence and phosphorescence of the crystal the vibronic emissive spectrum with FWHM 300 cm^–1^

the vibronic emissive spectrum with FWHM 400 cm^–1^

**Figure S4.** The observed and simulated emissive spectra at 298 K.

**Table S1.** The frequencies of the title compound according to QM and ONIOM calculations

|  | | | | In vacuum | | | In DCM | | | In cluster | | |
| --- | --- | --- | --- | --- | --- | --- | --- | --- | --- | --- | --- | --- |
| . | S_0_ state | | S_1_ state | | T_1_ state | S_0_ state | S_1_ state | T_1_ state | S_0_ state | | S_1_ state | T_1_ state |
| v_1_ | | 108.5 | 64.2 | | 66 | 107.2 | 70.2 | 63.8 | 150.5 | | 122.6 | 143.2 |
| v_2_ | | 108.7 | 95.6 | | 81.3 | 108.3 | 103.7 | 80.4 | 160.2 | | 143.6 | 145.9 |
| v_3_ | | 114.7 | 112.2 | | 97.8 | 117.3 | 111.1 | 99.4 | 172.7 | | 161.5 | 162.6 |
| v_4_ | | 250.5 | 213.5 | | 169.3 | 250.1 | 207.7 | 167.6 | 276.5 | | 244.9 | 209.1 |
| v_5_ | | 280.2 | 247 | | 256.9 | 281.7 | 250.2 | 257.6 | 288.7 | | 259.2 | 272.9 |
| v_6_ | | 280.3 | 280.4 | | 275.2 | 282.2 | 279.9 | 275.5 | 291.8 | | 284.9 | 281.2 |
| v_7_ | | 349.9 | 298.4 | | 282.5 | 352.9 | 297.9 | 284.3 | 366.3 | | 310.5 | 293.8 |
| v_8_ | | 349.9 | 313.9 | | 331.2 | 353.7 | 319.5 | 332.5 | 374.1 | | 321.4 | 336.8 |
| v_9_ | | 487.9 | 436.1 | | 443.6 | 488.2 | 437 | 444.3 | 491.1 | | 441.5 | 400.1 |
| v_10_ | | 487.9 | 461.8 | | 452.8 | 488.4 | 461.5 | 453.5 | 491.5 | | 468.3 | 436.9 |
| v_11_ | | 527.8 | 516.7 | | 477.6 | 528 | 514.9 | 476.9 | 533.1 | | 515.1 | 468.3 |
| v_12_ | | 615.8 | 551.8 | | 494.4 | 615.1 | 551 | 499.6 | 621.7 | | 528.1 | 480.8 |
| v_13_ | | 615.8 | 562.9 | | 534.1 | 615.5 | 562.2 | 538.8 | 630 | | 561.9 | 534.6 |
| v_14_ | | 632.1 | 580.4 | | 598.7 | 631.9 | 579.8 | 599 | 643.1 | | 577.9 | 571.5 |
| v_15_ | | 661.7 | 587.8 | | 611.2 | 661.1 | 584.1 | 611.7 | 663.7 | | 589.4 | 605.8 |
| v_16_ | | 678.9 | 634.6 | | 636.7 | 681.5 | 633.2 | 635.6 | 692.6 | | 619.1 | 615.6 |
| v_17_ | | 679.2 | 644 | | 654.9 | 682 | 636.5 | 656.2 | 695.1 | | 639.1 | 645.1 |
| v_18_ | | 684.5 | 664.3 | | 669.1 | 686.1 | 660.3 | 669.9 | 700.8 | | 661.1 | 675 |
| v_19_ | | 731.1 | 706.6 | | 683.2 | 728.9 | 705.9 | 683.7 | 736.2 | | 706.9 | 688 |
| v_20_ | | 732.1 | 714.2 | | 715.1 | 730.1 | 710.6 | 713.9 | 739.8 | | 716.4 | 748.1 |
| v_21_ | | 734.7 | 755 | | 732.7 | 734.4 | 763.5 | 731.5 | 770.1 | | 776.4 | 749.1 |
| v_22_ | | 857.3 | 854.1 | | 822 | 859.6 | 861.5 | 823.6 | 859.1 | | 864.7 | 826.7 |
| v_23_ | | 857.4 | 861.5 | | 842.1 | 860.1 | 867 | 844.5 | 873.8 | | 868.5 | 842.3 |
| v_24_ | | 857.6 | 872 | | 850 | 860.7 | 872.3 | 850 | 893.9 | | 878.2 | 862.9 |
| v_25_ | | 918.5 | 876.4 | | 865.3 | 918.4 | 876.9 | 866.4 | 921.3 | | 885.8 | 870.7 |
| v_26_ | | 919.3 | 884.3 | | 903.8 | 918.5 | 882.5 | 908.2 | 924.6 | | 891.9 | 912.4 |
| v_27_ | | 919.3 | 892.2 | | 907.6 | 919.6 | 889.9 | 910.1 | 926.1 | | 909.3 | 927.4 |
| v_28_ | | 945.3 | 917.7 | | 924.2 | 943.6 | 913 | 923.7 | 949.3 | | 923.6 | 934.4 |
| v_29_ | | 1070.5 | 1011.8 | | 972.8 | 1071.2 | 1016.4 | 975.6 | 1077.6 | | 1019 | 954.8 |
| v_30_ | | 1070.9 | 1030.3 | | 1039 | 1071.8 | 1028.7 | 1043 | 1080.5 | | 1039.4 | 1038.1 |
| v_31_ | | 1109.1 | 1056.2 | | 1049.4 | 1110.7 | 1056.4 | 1049 | 1119.1 | | 1065.4 | 1057.6 |
| v_32_ | | 1124.1 | 1085.7 | | 1095.5 | 1120.8 | 1081.9 | 1095.5 | 1129.4 | | 1086.7 | 1107.9 |
| v_33_ | | 1124.2 | 1094.4 | | 1107.9 | 1121.1 | 1092.7 | 1105.7 | 1132.2 | | 1105.2 | 1116 |
| v_34_ | | 1167.6 | 1108.4 | | 1114.7 | 1162.9 | 1110.1 | 1112.9 | 1173.2 | | 1118.2 | 1122.2 |
| v_35_ | | 1167.7 | 1157.8 | | 1137 | 1162.9 | 1157.2 | 1135 | 1174.6 | | 1167.8 | 1148.9 |
| v_36_ | | 1170.3 | 1176.9 | | 1157.3 | 1165.2 | 1169.7 | 1153.1 | 1176.7 | | 1185.3 | 1167 |
| v_37_ | | 1274.4 | 1197 | | 1199.9 | 1273.1 | 1194.6 | 1194.9 | 1275.7 | | 1208.7 | 1209.5 |
| v_38_ | | 1274.5 | 1201.2 | | 1240.5 | 1273.2 | 1213.2 | 1240.8 | 1276 | | 1221.1 | 1235.2 |
| v_39_ | | 1294.5 | 1243 | | 1262 | 1293.7 | 1238.6 | 1262.5 | 1301.5 | | 1250.9 | 1267.2 |
| v_40_ | | 1345.1 | 1264.5 | | 1317.6 | 1344.8 | 1264.3 | 1316.5 | 1347.8 | | 1271.4 | 1322 |
| v_41_ | | 1363.9 | 1297.2 | | 1332.2 | 1362 | 1282.3 | 1330.6 | 1370 | | 1326.5 | 1338.8 |
| v_42_ | | 1364.1 | 1323.8 | | 1341.6 | 1362.2 | 1320.4 | 1340.5 | 1370.5 | | 1331.2 | 1346.2 |
| v_43_ | | 1470.7 | 1364.5 | | 1393.1 | 1471 | 1363.1 | 1391.4 | 1475 | | 1370.2 | 1380.3 |
| v_44_ | | 1482.6 | 1387.5 | | 1412.4 | 1483.1 | 1384.7 | 1410.7 | 1479.3 | | 1396.3 | 1425.3 |
| v_45_ | | 1482.7 | 1432.5 | | 1456.5 | 1483.2 | 1427.4 | 1456.7 | 1481.9 | | 1437.1 | 1474.1 |
| v_46_ | | 1528.7 | 1476.7 | | 1501.1 | 1531.7 | 1471.2 | 1495.3 | 1526.3 | | 1478.5 | 1502 |
| v_47_ | | 1551.4 | 1487.7 | | 1532.7 | 1553.8 | 1489.5 | 1525.4 | 1549.3 | | 1488.6 | 1552.7 |
| v_48_ | | 1551.6 | 1524.8 | | 1554.9 | 1554 | 1518.7 | 1554.5 | 1550.8 | | 1532.9 | 1554.3 |
| v_49_ | | 1651.4 | 1588.2 | | 1559.7 | 1647.4 | 1576.9 | 1562.6 | 1639.8 | | 1587.2 | 1561.5 |
| v_50_ | | 1659.9 | 1650.7 | | 1648.8 | 1650.9 | 1626.8 | 1643.8 | 1652.6 | | 1661 | 1633.6 |
| v_51_ | | 1660.1 | 1710.7 | | 1658.9 | 1651.1 | 1687.4 | 1649.3 | 1654.1 | | 1712.3 | 1651.7 |
| v_52_ | | 3290.5 | 3231.8 | | 3236.1 | 3291.4 | 3228.4 | 3246.2 | 3309.3 | | 3267.3 | 3264 |
| v_53_ | | 3290.9 | 3265.5 | | 3287.8 | 3291.8 | 3273.7 | 3287.9 | 3321 | | 3291.8 | 3307.8 |
| v_54_ | | 3291.2 | 3271.1 | | 3292.1 | 3292.1 | 3276.9 | 3293.4 | 3326.6 | | 3316.7 | 3313.1 |
| v_55_ | | 3328.2 | 3308.2 | | 3305.3 | 3330.7 | 3311.1 | 3309.9 | 3340.5 | | 3335.6 | 3330.2 |
| v_56_ | | 3332.4 | 3312.8 | | 3331.3 | 3333.7 | 3314.4 | 3330.7 | 3341.8 | | 3340.8 | 3340.3 |
| v_57_ | | 3332.7 | 3317 | | 3331.5 | 3333.8 | 3319.1 | 3332.7 | 3359.1 | | 3349.2 | 3356.1 |
|  | |  |  | |  |  |  |  |  | |  |  |

**Table S2.** The reorganization energy (λ_reorg_/cm^–1^) and Huang–Rhys factor (HR) between S_1_ and T_1_ minima by NMA method with internal coordinate

|  | | S_1_→T_1_ in gas phase | | T_1_→S_1_ in gas phase | | S_1_→T_1_ in DCM | | T_1_→S_1_ in DCM | | S_1_→T_1_ in Cluster | | | T_1_→S_1_ in cluster | |
| --- | --- | --- | --- | --- | --- | --- | --- | --- | --- | --- | --- | --- | --- | --- |
| . | λ/cm^–1^ | | HR | λ/cm^–1^ | HR | λ/cm^–1^ | HR | λ/cm^–1^ | HR | λ/cm^–1^ | HR | λ/cm^–1^ | | HR |
| v_1_ | 506.1 | | 7.8764 | 447.4 | 6.7741 | 593.2 | 8.4492 | 908.6 | 14.2366 | 0 | 0.0002 | 481.3 | | 3.3611 |
| v_2_ | 58.7 | | 0.6138 | 56.4 | 0.6931 | 766.4 | 7.3879 | 593.7 | 7.3818 | 282.4 | 1.9661 | 841.3 | | 5.7652 |
| v_3_ | 511 | | 4.5525 | 519 | 5.3071 | 2592.4 | 23.3268 | 582.2 | 5.855 | 2025.6 | 12.538 | 36 | | 0.2216 |
| v_4_ | 421.6 | | 1.9744 | 4 | 0.0239 | 544.9 | 2.6227 | 24 | 0.1433 | 218.7 | 0.8927 | 0.4 | | 0.0022 |
| v_5_ | 243.6 | | 0.9861 | 7.2 | 0.0283 | 193.1 | 0.7716 | 930.4 | 3.6114 | 510.4 | 1.9685 | 628.1 | | 2.3011 |
| v_6_ | 580.4 | | 2.0698 | 401.7 | 1.4597 | 25.2 | 0.0903 | 27.3 | 0.0991 | 143.1 | 0.5021 | 3.1 | | 0.0113 |
| v_7_ | 3179.9 | | 10.6546 | 876.2 | 3.1012 | 3.4 | 0.0114 | 625.1 | 2.1987 | 15.5 | 0.05 | 129.1 | | 0.4396 |
| v_8_ | 1503 | | 4.7878 | 1628.6 | 4.916 | 803.4 | 2.5145 | 434.9 | 1.3076 | 524.3 | 1.631 | 1361.3 | | 4.0419 |
| v_9_ | 764 | | 1.7518 | 1391.4 | 3.1362 | 194.1 | 0.4442 | 749.6 | 1.6872 | 320.1 | 0.725 | 397 | | 0.9923 |
| v_10_ | 6 | | 0.013 | 2.1 | 0.0047 | 70.8 | 0.1536 | 402.3 | 0.8871 | 0 | 0 | 285.8 | | 0.6542 |
| v_11_ | 1641.4 | | 3.1763 | 0.4 | 0.0008 | 508.6 | 0.9877 | 0.3 | 0.0007 | 903.4 | 1.7536 | 680.6 | | 1.4533 |
| v_12_ | 31.7 | | 0.0575 | 1847.8 | 3.7372 | 3.4 | 0.0063 | 642.6 | 1.2861 | 2.5 | 0.0048 | 8.2 | | 0.017 |
| v_13_ | 396.9 | | 0.7052 | 3.6 | 0.0068 | 357.9 | 0.6367 | 7.7 | 0.0144 | 957.6 | 1.704 | 2.4 | | 0.0045 |
| v_14_ | 125.6 | | 0.2164 | 64 | 0.107 | 242.5 | 0.4183 | 313.2 | 0.5229 | 335.1 | 0.5799 | 162.1 | | 0.2837 |
| v_15_ | 178.1 | | 0.303 | 0.3 | 0.0005 | 165.1 | 0.2827 | 15.5 | 0.0254 | 104 | 0.1765 | 2.9 | | 0.0049 |
| v_16_ | 13.8 | | 0.0217 | 90.2 | 0.1417 | 1121.7 | 1.7713 | 76.1 | 0.1197 | 1631.9 | 2.6358 | 17.1 | | 0.0277 |
| v_17_ | 2058 | | 3.1956 | 3000 | 4.5808 | 330.4 | 0.5191 | 1949.6 | 2.9707 | 16.6 | 0.026 | 20.4 | | 0.0316 |
| v_18_ | 1930 | | 2.9051 | 154.4 | 0.2307 | 118.7 | 0.1798 | 1106.2 | 1.6513 | 108 | 0.1634 | 127 | | 0.1882 |
| v_19_ | 537.7 | | 0.7609 | 238.5 | 0.3491 | 1767.6 | 2.5038 | 377.5 | 0.5521 | 1550.3 | 2.1931 | 2255.3 | | 3.278 |
| v_20_ | 1777.5 | | 2.4888 | 21 | 0.0293 | 931.9 | 1.3114 | 601.4 | 0.8424 | 59.8 | 0.0835 | 65.4 | | 0.0874 |
| v_21_ | 94.9 | | 0.1257 | 306.7 | 0.4185 | 68 | 0.089 | 0.5 | 0.0007 | 7.1 | 0.0092 | 322.8 | | 0.431 |
| v_22_ | 18.3 | | 0.0214 | 9.3 | 0.0113 | 20.3 | 0.0235 | 188.9 | 0.2294 | 1.8 | 0.0021 | 0 | | 0 |
| v_23_ | 81.7 | | 0.0948 | 0.1 | 0.0001 | 2.4 | 0.0028 | 0 | 0 | 90.1 | 0.1038 | 8.4 | | 0.01 |
| v_24_ | 30.8 | | 0.0354 | 15.5 | 0.0182 | 217.3 | 0.2491 | 3.6 | 0.0043 | 0.4 | 0.0005 | 0.1 | | 0.0001 |
| v_25_ | 2.6 | | 0.003 | 1.3 | 0.0015 | 0.1 | 0.0002 | 57.7 | 0.0666 | 13.7 | 0.0154 | 33.6 | | 0.0386 |
| v_26_ | 360.6 | | 0.4078 | 93 | 0.1029 | 557.7 | 0.632 | 21.9 | 0.0241 | 163.3 | 0.1831 | 414.7 | | 0.4545 |
| v_27_ | 301.3 | | 0.3377 | 270.1 | 0.2976 | 23.8 | 0.0268 | 121.7 | 0.1338 | 37.2 | 0.0409 | 38.9 | | 0.042 |
| v_28_ | 19.6 | | 0.0214 | 1 | 0.0011 | 67.9 | 0.0744 | 157.9 | 0.1709 | 2.5 | 0.0027 | 12.8 | | 0.0138 |
| v_29_ | 12.7 | | 0.0125 | 185.5 | 0.1907 | 316.6 | 0.3114 | 382.5 | 0.3921 | 0 | 0 | 0.4 | | 0.0004 |
| v_30_ | 12.4 | | 0.0121 | 377.7 | 0.3635 | 231.6 | 0.2251 | 42.8 | 0.041 | 158.2 | 0.1522 | 518.1 | | 0.4991 |
| v_31_ | 19.3 | | 0.0182 | 129.2 | 0.1231 | 223.6 | 0.2116 | 240.3 | 0.2291 | 14.5 | 0.0136 | 15 | | 0.0142 |
| v_32_ | 1.5 | | 0.0014 | 1.2 | 0.0011 | 65.2 | 0.0603 | 143 | 0.1305 | 3.9 | 0.0035 | 97.1 | | 0.0876 |
| v_33_ | 849.9 | | 0.7765 | 21.7 | 0.0196 | 11.2 | 0.0103 | 27.9 | 0.0253 | 407.4 | 0.3686 | 20.8 | | 0.0186 |
| v_34_ | 40.1 | | 0.0362 | 252.3 | 0.2263 | 185.2 | 0.1668 | 195 | 0.1752 | 177.6 | 0.1588 | 16.7 | | 0.0149 |
| v_35_ | 132.3 | | 0.1143 | 47.6 | 0.0419 | 294.6 | 0.2545 | 81.5 | 0.0718 | 390.3 | 0.3342 | 33.3 | | 0.029 |
| v_36_ | 394.5 | | 0.3352 | 110.9 | 0.0958 | 12.6 | 0.0108 | 6.4 | 0.0056 | 139.6 | 0.1177 | 239.3 | | 0.205 |
| v_37_ | 0 | | 0 | 619 | 0.5159 | 377.2 | 0.3157 | 132.5 | 0.1108 | 545.5 | 0.4513 | 705.8 | | 0.5835 |
| v_38_ | 1697 | | 1.4127 | 1113.6 | 0.8977 | 53.6 | 0.0442 | 1554.2 | 1.2525 | 684.2 | 0.5603 | 507.4 | | 0.4107 |
| v_39_ | 179.2 | | 0.1442 | 682.1 | 0.5404 | 375.7 | 0.3033 | 59.9 | 0.0474 | 53.6 | 0.0429 | 379.4 | | 0.2994 |
| v_40_ | 0.6 | | 0.0005 | 626.6 | 0.4755 | 29.1 | 0.023 | 45.4 | 0.0345 | 56.3 | 0.0442 | 601 | | 0.4546 |
| v_41_ | 303.6 | | 0.234 | 172.9 | 0.1298 | 472.5 | 0.3684 | 43.9 | 0.0329 | 136.9 | 0.1032 | 89.6 | | 0.0669 |
| v_42_ | 490.2 | | 0.3702 | 1.2 | 0.0009 | 42.8 | 0.0324 | 8.3 | 0.0062 | 920.5 | 0.6915 | 0.7 | | 0.0005 |
| v_43_ | 378.5 | | 0.2773 | 800 | 0.5742 | 16.7 | 0.0122 | 127.8 | 0.0919 | 363.1 | 0.265 | 495.6 | | 0.3591 |
| v_44_ | 53.8 | | 0.0388 | 26.9 | 0.019 | 21.6 | 0.0156 | 143.8 | 0.1019 | 196.7 | 0.1409 | 46.4 | | 0.0325 |
| v_45_ | 160.9 | | 0.1123 | 88.7 | 0.0609 | 368.7 | 0.2583 | 1.6 | 0.0011 | 225.5 | 0.1569 | 78.7 | | 0.0534 |
| v_46_ | 3068.4 | | 2.0778 | 1345.8 | 0.8965 | 275.4 | 0.1871 | 996.6 | 0.6664 | 2819.3 | 1.9067 | 778.1 | | 0.518 |
| v_47_ | 613.5 | | 0.4124 | 1600.7 | 1.0444 | 174.9 | 0.1174 | 563.2 | 0.3692 | 5.9 | 0.0039 | 793.2 | | 0.5108 |
| v_48_ | 479.6 | | 0.3145 | 125.3 | 0.0806 | 14.1 | 0.0093 | 2.8 | 0.0018 | 157.6 | 0.1028 | 968.5 | | 0.6231 |
| v_49_ | 588.5 | | 0.3705 | 799.8 | 0.5128 | 0.1 | 0.0001 | 174.3 | 0.1115 | 770.1 | 0.4852 | 611.9 | | 0.3918 |
| v_50_ | 554.9 | | 0.3361 | 166.3 | 0.1008 | 27.2 | 0.0167 | 198.2 | 0.1205 | 160.7 | 0.0967 | 48.9 | | 0.0299 |
| v_51_ | 70.2 | | 0.041 | 0 | 0 | 997.9 | 0.5913 | 895.2 | 0.5427 | 122.5 | 0.0715 | 6.2 | | 0.0037 |
| v_52_ | 5.3 | | 0.0016 | 0.4 | 0.0001 | 6.2 | 0.0019 | 0.8 | 0.0002 | 0.7 | 0.0002 | 1.2 | | 0.0003 |
| v_53_ | 0 | | 0 | 0.2 | 0 | 1.6 | 0.0005 | 1 | 0.0003 | 0.4 | 0.0001 | 0.6 | | 0.0002 |
| v_54_ | 4.3 | | 0.0013 | 2 | 0.0006 | 0.8 | 0.0002 | 1.3 | 0.0004 | 1.8 | 0.0005 | 1.4 | | 0.0004 |
| v_55_ | 2.5 | | 0.0007 | 1.1 | 0.0003 | 0.3 | 0.0001 | 2.1 | 0.0006 | 0 | 0 | 6.1 | | 0.0018 |
| v_56_ | 0.3 | | 0.0001 | 0 | 0 | 2.2 | 0.0006 | 0.8 | 0.0002 | 4.3 | 0.0013 | 0.7 | | 0.0002 |
| v_57_ | 2.2 | | 0.0006 | 0.1 | 0 | 0.2 | 0 | 0.2 | 0 | 0.5 | 0.0001 | 0.2 | | 0 |
|  |  | |  |  |  |  |  |  |  |  |  |  | |  |

**Table S3.** The reorganization energy (λ_reorg_/cm^–1^), Huang–Rhys factor (HR) and nonadiabatic coupling matrix element between S_0_ and S_1_ minima of the title compound by NMA method with internal coordinate in gas phase and in DCM

|  | S_0_→S_1_ In gas phase | | | S_1_→S_0_ In gas phase | | | S_0_→S_1_ In DCM | | | | | | | S_1_→S_0_ In DCM | | |
| --- | --- | --- | --- | --- | --- | --- | --- | --- | --- | --- | --- | --- | --- | --- | --- | --- |
| . | λ/cm^–1^ | HR | R/cm^–1^ | | λ_reorg_ /cm^–1^ | HR | | λ_reorg_/cm^–1^ | | HR | | λ_reorg_/cm^–1^ | | | HR | R/cm^–1^ |
| v_1_ | 289.6 | 2.6681 | 2.62 | | 227.8 | 3.5458 | | | 471.1 | 4.394 | | | 195.1 | | 2.7797 | 0.9 |
| v_2_ | 229.7 | 2.112 | 3.87 | | 87 | 0.9099 | | | 1.5 | 0.0138 | | | 25.7 | | 0.2479 | 0.3 |
| v_3_ | 1103.7 | 9.6171 | 8.11 | | 1765.5 | 15.726 | | | 921.5 | 7.8522 | | | 1622.6 | | 14.6008 | 8.4 |
| v_4_ | 285.1 | 1.1382 | 3.14 | | 1.9 | 0.0091 | | | 280.5 | 1.1216 | | | 0.1 | | 0.0005 | 2.9 |
| v_5_ | 79.3 | 0.2831 | 0.32 | | 29.4 | 0.1191 | | | 23.8 | 0.0845 | | | 38.4 | | 0.1536 | 0.4 |
| v_6_ | 69.4 | 0.2479 | 0.07 | | 149.9 | 0.5347 | | | 128.5 | 0.4552 | | | 121.8 | | 0.4353 | 0 |
| v_7_ | 1945.4 | 5.5598 | 2.97 | | 819.7 | 2.7465 | | | 208.6 | 0.5912 | | | 678.5 | | 2.2772 | 0.2 |
| v_8_ | 1 | 0.003 | 0.4 | | 0.9 | 0.003 | | | 1696.3 | 4.7956 | | | 0.4 | | 0.0012 | 2.6 |
| v_9_ | 268.8 | 0.551 | 0.09 | | 169.1 | 0.3878 | | | 30.4 | 0.0622 | | | 147.8 | | 0.3382 | 0.2 |
| v_10_ | 1.7 | 0.0036 | 0.07 | | 2 | 0.0044 | | | 226.5 | 0.4637 | | | 1.5 | | 0.0033 | 1.5 |
| v_11_ | 450.1 | 0.8529 | 811.68 | | 293.7 | 0.5684 | | | 408.4 | 0.7734 | | | 338.7 | | 0.6578 | 1028.4 |
| v_12_ | 4 | 0.0066 | 0.35 | | 193.7 | 0.351 | | | 47.6 | 0.0774 | | | 195.9 | | 0.3556 | 0.3 |
| v_13_ | 52.4 | 0.0851 | 1.87 | | 298.6 | 0.5304 | | | 0.4 | 0.0007 | | | 262 | | 0.4661 | 0.1 |
| v_14_ | 4.5 | 0.0072 | 2.99 | | 168.5 | 0.2903 | | | 7.4 | 0.0118 | | | 299.1 | | 0.5159 | 3.6 |
| v_15_ | 58 | 0.0877 | 643 | | 250.3 | 0.4258 | | | 65.6 | 0.0993 | | | 217 | | 0.3715 | 667.5 |
| v_16_ | 518.6 | 0.7639 | 1 | | 0 | 0 | | | 979.3 | 1.4368 | | | 2178.1 | | 3.4395 | 1.6 |
| v_17_ | 652.7 | 0.961 | 0.16 | | 3348.6 | 5.1997 | | | 136.2 | 0.1997 | | | 778.6 | | 1.223 | 1.2 |
| v_18_ | 458.4 | 0.6697 | 1.23 | | 538.5 | 0.8106 | | | 421.2 | 0.6139 | | | 268.6 | | 0.4068 | 1.3 |
| v_19_ | 163.7 | 0.2239 | 0.79 | | 10 | 0.0142 | | | 188.6 | 0.2587 | | | 65.9 | | 0.0934 | 0.6 |
| v_20_ | 208.7 | 0.285 | 0.03 | | 390 | 0.546 | | | 224.8 | 0.3079 | | | 170 | | 0.2393 | 0.6 |
| v_21_ | 10.7 | 0.0146 | 1.07 | | 52.7 | 0.0699 | | | 80.6 | 0.1098 | | | 33.9 | | 0.0444 | 1.3 |
| v_22_ | 0.1 | 0.0001 | 0.1 | | 0.2 | 0.0003 | | | 0 | 0 | | | 177.1 | | 0.2055 | 0.1 |
| v_23_ | 9.2 | 0.0107 | 0.12 | | 207.7 | 0.2411 | | | 9.3 | 0.0109 | | | 2.4 | | 0.0028 | 0 |
| v_24_ | 17.2 | 0.0201 | 0.53 | | 50.7 | 0.0581 | | | 12.8 | 0.0149 | | | 88.4 | | 0.1013 | 0.3 |
| v_25_ | 243.5 | 0.2651 | 197.16 | | 29.9 | 0.0341 | | | 13.3 | 0.0145 | | | 2.6 | | 0.003 | 0.7 |
| v_26_ | 223.5 | 0.2432 | 2.69 | | 175.1 | 0.198 | | | 202.2 | 0.2202 | | | 100 | | 0.1134 | 14.5 |
| v_27_ | 3.3 | 0.0035 | 1.12 | | 36.7 | 0.0411 | | | 277 | 0.3012 | | | 5 | | 0.0056 | 210.6 |
| v_28_ | 16.5 | 0.0174 | 910.59 | | 178.3 | 0.1943 | | | 21.6 | 0.0229 | | | 166.6 | | 0.1824 | 990.8 |
| v_29_ | 15.3 | 0.0143 | 0.15 | | 29.9 | 0.0296 | | | 17.3 | 0.0161 | | | 46.9 | | 0.0462 | 1.7 |
| v_30_ | 0 | 0 | 0.22 | | 102.5 | 0.0995 | | | 0.8 | 0.0008 | | | 78.1 | | 0.0759 | 1.3 |
| v_31_ | 153.1 | 0.138 | 146.93 | | 17.8 | 0.0168 | | | 135.2 | 0.1217 | | | 9.7 | | 0.0092 | 160.7 |
| v_32_ | 9.4 | 0.0083 | 0.64 | | 95.4 | 0.0878 | | | 0 | 0 | | | 73.4 | | 0.0679 | 3.1 |
| v_33_ | 96.2 | 0.0856 | 0.73 | | 822.5 | 0.7515 | | | 74.3 | 0.0663 | | | 616.2 | | 0.5639 | 4.3 |
| v_34_ | 165.5 | 0.1418 | 50.95 | | 8.2 | 0.0074 | | | 23.5 | 0.0202 | | | 1.5 | | 0.0013 | 116.7 |
| v_35_ | 20.6 | 0.0176 | 8.89 | | 24.7 | 0.0213 | | | 150.6 | 0.1295 | | | 47.4 | | 0.041 | 95.5 |
| v_36_ | 92.1 | 0.0787 | 2220.08 | | 6.8 | 0.0058 | | | 119.2 | 0.1023 | | | 2.9 | | 0.0025 | 2270.8 |
| v_37_ | 406.3 | 0.3188 | 2.3 | | 170.1 | 0.1421 | | | 455.5 | 0.3577 | | | 22.9 | | 0.0191 | 1.5 |
| v_38_ | 277.5 | 0.2177 | 1.63 | | 543.6 | 0.4525 | | | 166.1 | 0.1305 | | | 479 | | 0.3948 | 5 |
| v_39_ | 46.6 | 0.036 | 1497.05 | | 109.1 | 0.0877 | | | 69.9 | 0.054 | | | 122.4 | | 0.0988 | 1424 |
| v_40_ | 13.8 | 0.0103 | 769.24 | | 28 | 0.0222 | | | 0.5 | 0.0003 | | | 0.2 | | 0.0001 | 756.3 |
| v_41_ | 120.7 | 0.0885 | 2.39 | | 253 | 0.1951 | | | 117.4 | 0.0862 | | | 500.3 | | 0.3901 | 3.7 |
| v_42_ | 27.6 | 0.0202 | 2.79 | | 0.1 | 0 | | | 19.9 | 0.0146 | | | 2.5 | | 0.0019 | 7.9 |
| v_43_ | 10.1 | 0.0069 | 654.75 | | 79.3 | 0.0581 | | | 4.1 | 0.0027 | | | 92.6 | | 0.0679 | 719.1 |
| v_44_ | 226.6 | 0.1528 | 3.66 | | 1.2 | 0.0008 | | | 612.5 | 0.413 | | | 0.2 | | 0.0001 | 7.2 |
| v_45_ | 446.5 | 0.3011 | 4.56 | | 19.2 | 0.0134 | | | 14.3 | 0.0096 | | | 15.1 | | 0.0106 | 2.7 |
| v_46_ | 505.1 | 0.3304 | 2505.7 | | 947.6 | 0.6416 | | | 549.6 | 0.3588 | | | 944.7 | | 0.6421 | 2530.4 |
| v_47_ | 9.4 | 0.006 | 3.56 | | 219 | 0.1472 | | | 27.2 | 0.0175 | | | 52.6 | | 0.0353 | 1.8 |
| v_48_ | 247.3 | 0.1593 | 11.92 | | 35.8 | 0.0235 | | | 275.9 | 0.1775 | | | 0.4 | | 0.0002 | 16.4 |
| v_49_ | 148.5 | 0.0899 | 538.87 | | 3.6 | 0.0023 | | | 124.7 | 0.0756 | | | 9.4 | | 0.006 | 472.9 |
| v_50_ | 108.1 | 0.0651 | 4.96 | | 1112 | 0.6736 | | | 271.1 | 0.1642 | | | 1048.5 | | 0.6445 | 5.6 |
| v_51_ | 599.1 | 0.3609 | 3.28 | | 1666.7 | 0.9742 | | | 286.8 | 0.1737 | | | 1110.3 | | 0.6579 | 10.1 |
| v_52_ | 0.4 | 0.0001 | 5.59 | | 5.6 | 0.0017 | | | 0.6 | 0.0002 | | | 5.9 | | 0.0018 | 6.8 |
| v_53_ | 1.1 | 0.0003 | 6.82 | | 0 | 0 | | | 1.3 | 0.0004 | | | 1.6 | | 0.0004 | 7.9 |
| v_54_ | 0.7 | 0.0002 | 7.2 | | 2.2 | 0.0006 | | | 0.7 | 0.0002 | | | 0 | | 0 | 8 |
| v_55_ | 0 | 0 | 39.98 | | 1.3 | 0.0004 | | | 0 | 0 | | | 0 | | 0 | 39.9 |
| v_56_ | 0.5 | 0.0001 | 43.76 | | 0 | 0 | | | 0.1 | 0 | | | 0.8 | | 0.0002 | 53.5 |
| v_57_ | 0.1 | 0 | 32.24 | | 0 | 0 | | | 0.3 | 0.0001 | | | 0.1 | | 0 | 3.3 |
|  |  |  |  | |  |  | | |  | |  | |  | |  |  |

**Table S4.** The reorganization energy (λ_reorg_ /cm^–1^), Huang–Rhys factor (HR), nonadiabatic coupling matrix element and Franck–Condon factor (FC) between S_0_ and S_1_ minima of the title compound by NMA method with internal coordinate in cluster

|  | | S_0_→S_1_ In cluster | | |  | | S_1_→S_0_ In cluster | | |
| --- | --- | --- | --- | --- | --- | --- | --- | --- | --- |
| . | λ/cm^–1^ | | HR | R | | λ_reorg_/cm^–1^ | | HR | FC |
| v_1_ | 285.8 | | 1.8981 | 28.2 | | 12.4 | | 0.1017 | 0.0004 |
| v_2_ | 487.8 | | 3.0442 | 225.6 | | 193.2 | | 1.3452 | 0.0215 |
| v_3_ | 37.1 | | 0.215 | 351.9 | | 1008 | | 6.2397 | 0.0000 |
| v_4_ | 118.9 | | 0.43 | 5.3 | | 5.3 | | 0.0218 | 0.0000 |
| v_5_ | 151.6 | | 0.5252 | 10.7 | | 103.4 | | 0.3988 | 0.0096 |
| v_6_ | 26.4 | | 0.0906 | 43.9 | | 151.8 | | 0.5327 | 0.0153 |
| v_7_ | 1281.9 | | 3.4993 | 201.6 | | 459.9 | | 1.4812 | 0.0191 |
| v_8_ | 48.8 | | 0.1304 | 5.3 | | 29.9 | | 0.0932 | 0.0003 |
| v_9_ | 18.3 | | 0.0374 | 62.3 | | 81.7 | | 0.1851 | 0.0018 |
| v_10_ | 221.6 | | 0.4508 | 2.3 | | 0.3 | | 0.0007 | 0.0000 |
| v_11_ | 304 | | 0.5703 | 825 | | 428 | | 0.8309 | 0.0237 |
| v_12_ | 11.6 | | 0.0186 | 58.7 | | 2.8 | | 0.0054 | 0.0000 |
| v_13_ | 6.5 | | 0.0103 | 95.2 | | 1129.8 | | 2.0104 | 0.0098 |
| v_14_ | 4.9 | | 0.0076 | 32.8 | | 256.9 | | 0.4445 | 0.0116 |
| v_15_ | 63.2 | | 0.0953 | 635.2 | | 0 | | 0 | 0.0000 |
| v_16_ | 1475.6 | | 2.1305 | 42.9 | | 1815.1 | | 2.9317 | 0.0019 |
| v_17_ | 389.4 | | 0.5601 | 199.9 | | 11.3 | | 0.0178 | 0.0000 |
| v_18_ | 130.5 | | 0.1862 | 58.8 | | 61.1 | | 0.0924 | 0.0003 |
| v_19_ | 25.8 | | 0.035 | 33.7 | | 3.7 | | 0.0053 | 0.0000 |
| v_20_ | 544.1 | | 0.7355 | 5.1 | | 64.1 | | 0.0894 | 0.0003 |
| v_21_ | 19.3 | | 0.0251 | 7.9 | | 7 | | 0.0091 | 0.0000 |
| v_22_ | 25.2 | | 0.0293 | 22.1 | | 0.6 | | 0.0008 | 0.0000 |
| v_23_ | 2.9 | | 0.0033 | 26.9 | | 145.4 | | 0.1674 | 0.0014 |
| v_24_ | 3.7 | | 0.0042 | 48.3 | | 171.1 | | 0.1948 | 0.0021 |
| v_25_ | 109.8 | | 0.1192 | 311.7 | | 34.8 | | 0.0393 | 0.0000 |
| v_26_ | 277 | | 0.2996 | 29.4 | | 46.3 | | 0.0519 | 0.0001 |
| v_27_ | 1.5 | | 0.0016 | 41.5 | | 1.4 | | 0.0015 | 0.0000 |
| v_28_ | 10.3 | | 0.0108 | 844.1 | | 65.4 | | 0.0708 | 0.0001 |
| v_29_ | 9.4 | | 0.0087 | 30.5 | | 9.2 | | 0.0091 | 0.0000 |
| v_30_ | 13.6 | | 0.0126 | 23 | | 12 | | 0.0115 | 0.0000 |
| v_31_ | 92.1 | | 0.0823 | 67.1 | | 12.6 | | 0.0118 | 0.0000 |
| v_32_ | 31 | | 0.0274 | 4.8 | | 79.4 | | 0.0731 | 0.0002 |
| v_33_ | 54.7 | | 0.0483 | 94.5 | | 445.6 | | 0.4032 | 0.0098 |
| v_34_ | 20 | | 0.017 | 595.6 | | 34.4 | | 0.0308 | 0.0000 |
| v_35_ | 249.4 | | 0.2123 | 1379.8 | | 4 | | 0.0034 | 0.0000 |
| v_36_ | 16.5 | | 0.014 | 1726.8 | | 3.5 | | 0.0029 | 0.0000 |
| v_37_ | 99.5 | | 0.078 | 99 | | 38.7 | | 0.032 | 0.0000 |
| v_38_ | 553.7 | | 0.4339 | 27.3 | | 556.4 | | 0.4556 | 0.0121 |
| v_39_ | 18.1 | | 0.0139 | 1393.7 | | 82.8 | | 0.0662 | 0.0001 |
| v_40_ | 9.4 | | 0.007 | 784.3 | | 3.3 | | 0.0026 | 0.0000 |
| v_41_ | 74 | | 0.054 | 29.3 | | 22.5 | | 0.0169 | 0.0000 |
| v_42_ | 21.6 | | 0.0158 | 6 | | 309.4 | | 0.2324 | 0.0031 |
| v_43_ | 0.2 | | 0.0001 | 590.4 | | 85.7 | | 0.0625 | 0.0001 |
| v_44_ | 562.2 | | 0.38 | 74.7 | | 41.5 | | 0.0297 | 0.0000 |
| v_45_ | 54.4 | | 0.0367 | 169.4 | | 43.4 | | 0.0302 | 0.0000 |
| v_46_ | 455.4 | | 0.2983 | 2487.5 | | 947.9 | | 0.641 | 0.0192 |
| v_47_ | 224.3 | | 0.1447 | 102.5 | | 4.5 | | 0.003 | 0.0000 |
| v_48_ | 55.6 | | 0.0358 | 71.5 | | 23 | | 0.015 | 0.0000 |
| v_49_ | 113.2 | | 0.069 | 470.9 | | 3.3 | | 0.002 | 0.0000 |
| v_50_ | 222.3 | | 0.1345 | 31.1 | | 1027.1 | | 0.6183 | 0.0185 |
| v_51_ | 477.8 | | 0.2888 | 149.5 | | 1274.7 | | 0.7444 | 0.0221 |
| v_52_ | 0.9 | | 0.0003 | 3.5 | | 1 | | 0.0003 | 0.0000 |
| v_53_ | 0.4 | | 0.0001 | 18.3 | | 0.3 | | 0.0001 | 0.0000 |
| v_54_ | 0.7 | | 0.0002 | 3.2 | | 1.1 | | 0.0003 | 0.0000 |
| v_55_ | 0 | | 0 | 39.3 | | 0 | | 0 | 0.0000 |
| v_56_ | 0.3 | | 0.0001 | 40.2 | | 0 | | 0 | 0.0000 |
| v_57_ | 0.7 | | 0.0002 | 35.5 | | 1 | | 0.0003 | 0.0000 |
|  |  | |  |  | |  | |  |  |
